# High Potential for Using DNA from Ancient Herring Bones to Inform Modern Fisheries Management and Conservation

**DOI:** 10.1371/journal.pone.0051122

**Published:** 2012-11-30

**Authors:** Camilla F. Speller, Lorenz Hauser, Dana Lepofsky, Jason Moore, Antonia T. Rodrigues, Madonna L. Moss, Iain McKechnie, Dongya Y. Yang

**Affiliations:** 1 Ancient DNA Laboratory, Department of Archaeology, Simon Fraser University, Burnaby, British Columbia, Canada; 2 Department of Archaeology, University of York, University of York, Heslington, York, United Kingdom; 3 School of Aquatic and Fishery Sciences, University of Washington, Seattle, Washington, United States of America; 4 Department of Archaeology, Simon Fraser University, Burnaby, British Columbia, Canada; 5 Department of Anthropology, University of Oregon, Eugene, Oregon, United States of America; 6 Department of Anthropology, University of British Columbia, Vancouver, British Columbia, Canada; Natural History Museum of Denmark, University of Copenhagen, Denmark

## Abstract

Pacific herring (*Clupea pallasi*) are an abundant and important component of the coastal ecosystems for the west coast of North America. Current Canadian federal herring management assumes five regional herring populations in British Columbia with a high degree of exchange between units, and few distinct local populations within them. Indigenous traditional knowledge and historic sources, however, suggest that locally adapted, distinct regional herring populations may have been more prevalent in the past. Within the last century, the combined effects of commercial fishing and other anthropogenic factors have resulted in severe declines of herring populations, with contemporary populations potentially reflecting only the remnants of a previously more abundant and genetically diverse metapopulation. Through the analysis of 85 archaeological herring bones, this study attempted to reconstruct the genetic diversity and population structure of ancient herring populations using three different marker systems (mitochondrial DNA (mtDNA), microsatellites and SNPs). A high success rate (91%) of DNA recovery was obtained from the extremely small herring bone samples (often <10 mg). The ancient herring mtDNA revealed high haplotype diversity comparable to modern populations, although population discrimination was not possible due to the limited power of the mtDNA marker. Ancient microsatellite diversity was also similar to modern samples, but the data quality was compromised by large allele drop-out and stuttering. In contrast, SNPs were found to have low error rates with no evidence for deviations from Hardy-Weinberg equilibrium, and simulations indicated high power to detect genetic differentiation if loci under selection are used. This study demonstrates that SNPs may be the most effective and feasible approach to survey genetic population structure in ancient remains, and further efforts should be made to screen for high differentiation markers.This study provides the much needed foundation for wider scale studies on temporal genetic variation in herring, with important implications for herring fisheries management, Aboriginal title rights and herring conservation.

## Introduction

In a world of rapidly eroding biological diversity, maintaining and restoring healthy marine ecosystems have become a top priority for most resource managers and coastal communities. Setting effective targets for management action can be extremely difficult, however, without a clear understanding of appropriate ecological baselines [1]. When faced with a lack of long-term data on ecosystem dynamics, management targets are often based on retroactive applications of modern data or on information collected in the relatively recent past, both of which potentially represent ecological baselines far removed from actual pre-industrial conditions [2]. Resource managers are increasingly turning to archaeological data and indigenous traditional ecological knowledge to provide a long-term perspective on pre-industrial ecological states (e.g.[3]–[7]) and ecologically and culturally salient baselines for conservation (e.g. [8]).

The modern management and conservation of Pacific herring (*Clupea pallasi*) is one area which can benefit from such a long-term perspective. Archaeological and ethnobiological research indicate that Pacific herring were an abundant and important component of the coastal ecosystems of western North America [9]–[13]. Herring on the west coast of Canada and the contiguous US have been exploited by industrial fisheries for more than a century, with periods of depletion occurring at least in the 1930s and the 1960s [14], [15]. Data on British Columbia herring populations and spawning behavior have only been recorded since the 1940s, however, historic sources suggest that significant anthropogenic impacts on Pacific herring had already taken place as early as the 1880s and 90s in British Columbia [15] and after the turn of the 20^th^ century in Alaska [16].

Current Canadian federal herring management practices assume five regional herring populations in British Columbia (occurring in the Strait of Georgia, the west coast of Vancouver Island, the central coast, the north coast, and the southeastern coast of the Queen Charlotte Islands), with a high degree of exchange between spawning grounds within each unit [17]. This management strategy is supported at a coarse scale by genetic analyses of modern populations (collected in the 1990s and 2000s), which indicate high levels of genetic diversity within each region and limited phylogeographic patterning of herring stocks [18], [19]. While mtDNA analyses of Pacific herring have revealed genetic distinctions between Bering Sea and northeast Pacific populations of Pacific herring, no phylogeographic apportioning of herring lineages could be observed within British Columbia's stocks [19], [20]. While more powerful microsatellite analyses conducted by Beacham et al. [18] noted low differentiation among the five provincial herring stocks, they did identify distinct mainland inlet spawners and late spawning populations within these larger management groupings [18]. Other microsatellite studies have also identified population structuring in certain late spawning or geographically isolated herring populations in Alaska, British Columbia and Washington [18], [21]–[23], indicating detectable population structure among genetically distinct local populations. In addition to the large migratory stocks, many parts of the coast host 'resident' herring stocks (i.e., those herring that do not migrate great distances and display relatively high levels of spawning site fidelity) [24]–[26]. It is unknown to what degree herring migration is affected by trophic or climatic conditions, and to what extent these non-migratory herring may represent genetically differentiated stocks.

Indigenous traditional knowledge and historic sources support the hypothesis that locally adapted, distinct herring populations may have been more prevalent in the past than today. Coastal First Nations report that herring consistently returned to the same bays to spawn every year and the archaeological record demonstrates consistency in spawning locations through the millennia (McKechnie, Lepofsky, and Moss unpublished data). In addition, historical sources note that reduction fisheries of the late 1800s led to the disappearance of substantial herring aggregations from Burrard Inlet in southeastern Georgia Strait and other bays beginning in the early 1900s [15]; these presumably were local populations. Many locations on the coast exhibit a consistent archaeological record of herring, but do not support herring populations today (McKechnie, Lepofsky, and Moss, unpublished data). Considering the cumulative forage fish biomass removed over the last century and widespread archaeological abundance, it seems likely that contemporary populations are remnants of a previously more abundant and much more diverse metapopulation. The historic declines in herring numbers and the possible truncation of genetic diversity have uncertain consequences for overall species abundance, stability, and resilience.

Ancient DNA analysis provides a new approach for recognizing temporal shifts in biocomplexity, including reductions in overall species abundance and genetic diversity [27], [28]. Ancient DNA analysis of archaeological remains allows for a direct comparison of contemporary and long-term historical patterns of genetic variability, and the ability to explicitly quantify temporal changes in population-level diversity. Ancient DNA techniques have been successful at reconstructing the long-term population dynamics of terrestrial mammals such as musk-ox, cave bear [29], brown bear [30], wooly mammoth [31], steppe bison [32], [33], North American horse [34] and South American tuco-tuco [35]. Applications of ancient DNA in teleost populations are rarer, but analyses of ancient European sturgeon remains have been used to distinguish species, identify hybridization and estimate founding population sizes [36], [37]. In African catfish, ancient DNA analyses were used to reconstruct Roman trade routes in Turkey [38]. In the northeast Pacific, several studies have employed ancient DNA to identify the species of salmon exploited at coastal archaeological sites [39]–[41]. Most of these studies targeted mtDNA which occurs in multiple copies per cell and is therefore easier to amplify but has limited power to resolve localized population structure in high gene-flow species [42]. The scope of population genetic studies in marine fish based on multiple nuclear loci (e.g. microsatellites, single nucleotide polymorphisms (SNPs)), although highly informative of temporal changes in diversity and structure, has been limited to decade-old scale samples [43], [44].

One study ascertaining the potential for recovering microsatellites from historic herring scales has been attempted [45]. To date, no studies have ascertained the possibility of extracting well preserved DNA from individual archaeological herring bones dating back hundreds to thousands of years and weighing as little as 3 mg – significantly less than the quantity of material typically extracted in ancient DNA studies. Through the extraction and analysis of genetic material from archaeological herring remains, ancient DNA analysis has the capability to identify changes in herring biocomplexity through time and to test the hypothesis whether there were more distinct regional herring populations in the past.

**Figure 1:**
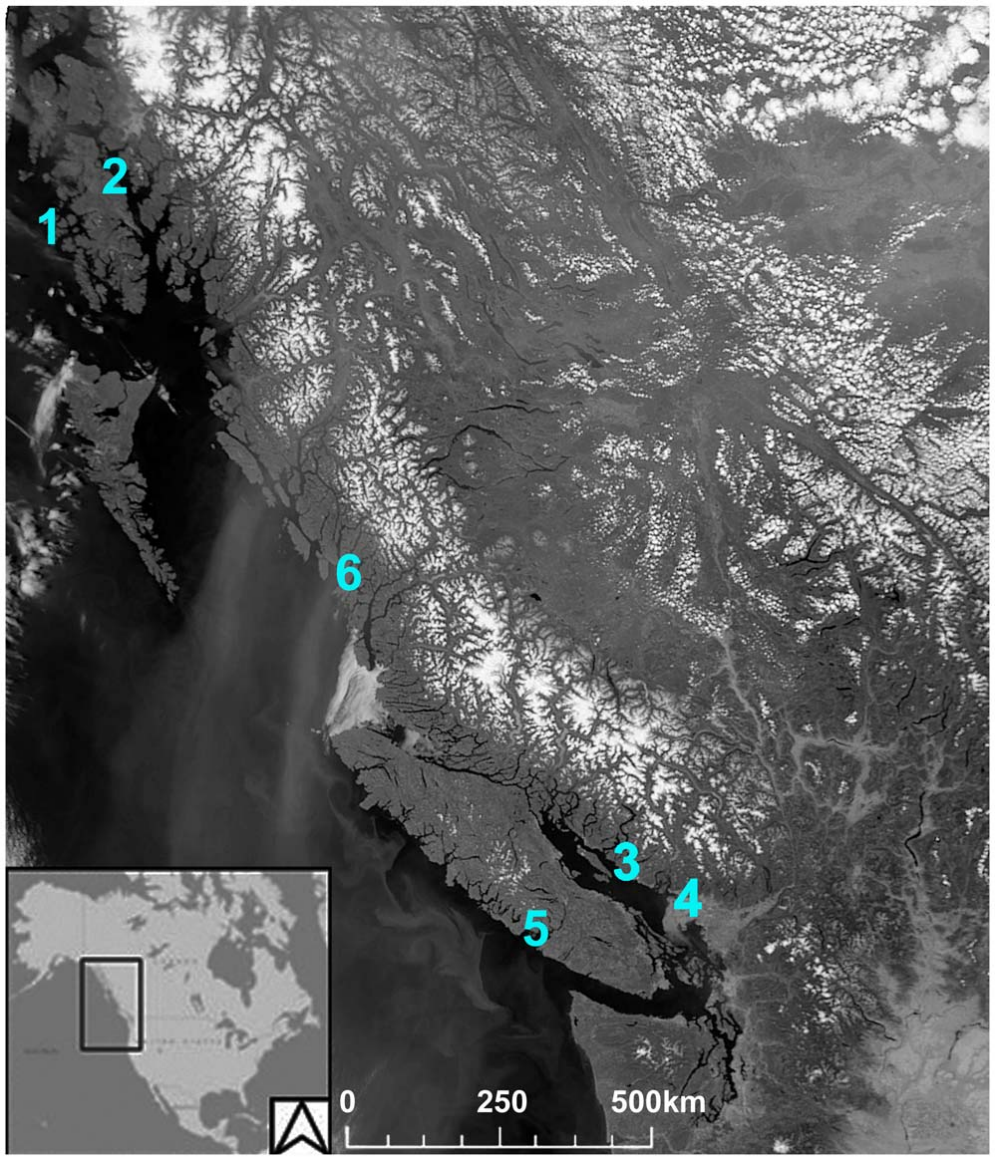
Approximate locations of archaeological sites from which herring bones were recovered and analyzed in this study. (1) Cape Addington Rockshelter (Southeast Alaska), (2) the Coffman Cove Site, and the Coffman Cove Ferry Terminal (Southeast Alaska); (3) Klehkwahnnohm and Kahkeeky (northern Georgia Strait); (4) Tum-tumay-whueton (Burrard Inlet, southeastern Georgia Strait); (5) Broken Group Islands, Barkley Sound (west coast of Vancouver Island). Modern samples analyzed in this study were obtained from the Central Coast of British Columbia (6).

The aims of this study were fourfold: first, we applied ancient DNA techniques to 85 archaeological herring samples recovered from four regions of British Columbia and Alaska (Table S1, Fig. 1) to test whether mitochondrial and nuclear DNA could be successfully extracted and amplified from individual archaeological herring skeletal elements. Second, we compared genetic diversity and differentiation of archaeological herring samples with those from contemporary populations at two mtDNA fragments (cytochrome b (cytb) and D-loop). Given the low spatial differentiation of mtDNA [19], [20], [46], our expectation was to find little if any temporal genetic differentiation. Third, we compiled preliminary data on amplification success and conformance to population genetic expectations in microsatellites and SNPs to ascertain the potential of using more powerful multilocus nuclear marker systems for estimating genetic diversity and population structure. Finally, we compared the power of mtDNA, microsatellites and SNPs to detect true temporal and spatial genetic differentiation by simulations based on empirical data. Our results provide the necessary foundation for wider scale studies on temporal genetic variation in herring which could address issues as diverse as climate change impacts, verification of commonly used but untested population genetic models, investigation of industrial fishery impacts, and First Nation herring management practices.

## Results

### Mitochondrial DNA

PCR amplifications of mtDNA were successful in 88% of the archaeological herring bones (75 of the 85 samples) for both cytb and D-loop (Genbank Accessions JX013703-JX013849). Amplifications failed for both markers in eight samples, and for one of the two markers in two additional samples. Successful PCR amplifications and clear sequences were obtained from archaeological vertebrae weighing as little as 3 mg (Fig. S1). Damage derived and polymerase enzyme misincorporations were observed in only 10 samples (6.6% of the sequences overall). Nine instances of ‘Type 2’ transitions (C→T/G→A) were observed in seven samples, and are likely the result of cytosine deamination [47]–[49]. Four ‘Type 1’ transitions were observed in three samples (A→G in all cases) which may be the result of polymerase misincorporation [47]. No significant differences were observed in PCR amplification success rate based on the age of the sample (chi-square with Yates’ correction for continuity, *χ^2^* = 2.815, p = 0.421), with successful amplification of samples dating to 3800 cal BP. Phylogenetic trees of both cytb and D-loop haplotypes and select *Clupea* reference sequences from Genbank [19], [47] illustrated that the ancient haplotypes group with *C. pallasi* sequences (Figure S2), confirming the species identification of Pacific herring for all successfully amplified samples. Additionally, the cytb sequences demonstrate large scale phylogeographic patterning, separating Eastern Pacific haplotypes (clade CP1) from those predominantly found in Western Pacific and Bering Sea herring populations (clade CP2) (Figure S2).

**Figure 2:**
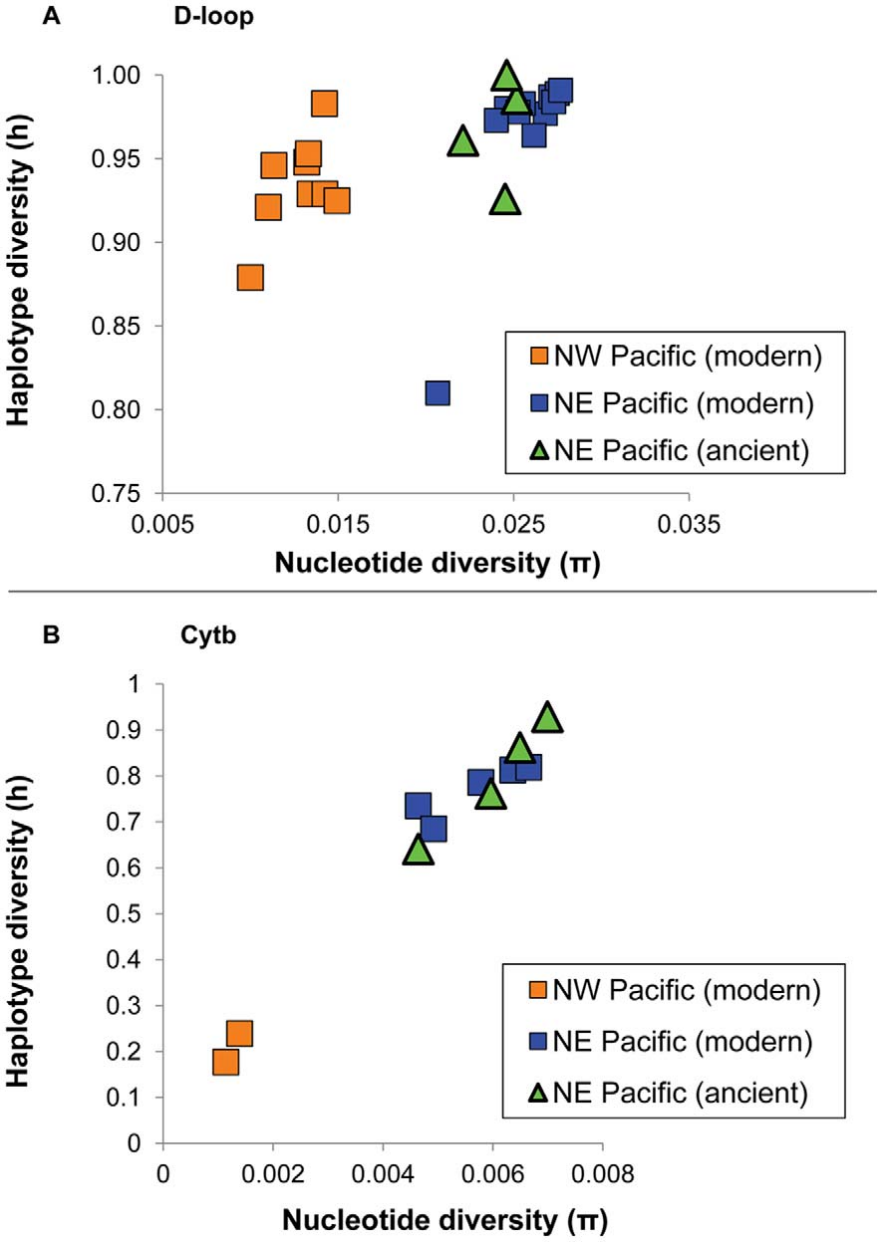
Plots of genetic diversity. Graphs displaying genetic diversity (nucleotide diversity and haplotype diversity) of samples from the two major phylogeographic groups described in Liu et al [19], and the ancient DNA samples from British Columbia and Alaska, for A) D-loop (above) and B) cytb (below). Note the lower diversity of the northwest Pacific group and the close clustering of ancient samples with the modern samples from the northeast Pacific. The one outlying sample with low D-loop haplotype diversity is from Portage Inlet (southern Georgia Strait), which shows similar cytb diversity to the other samples.

Genetic diversity in the ancient samples was similar to that observed in modern herring [19]. Nineteen different cytb haplotypes, and 42 different D-loop haplotypes were identified in the ancient material (Table S1). Comparison with modern Pacific herring sequence data [19] trimmed to the same length as ancient samples, showed lower genetic diversity in northwestern Pacific and Bering Sea herring compared to northeastern Pacific samples, though the difference was much more pronounced in cytb (Fig. 2, Tables S2 and S3). Haplotype and nucleotide diversity of both mtDNA regions were similar between ancient and modern samples collected from the same northeastern Pacific phylogeographic group (Fig. 2, Table S2 and S3).

**Figure 3:**
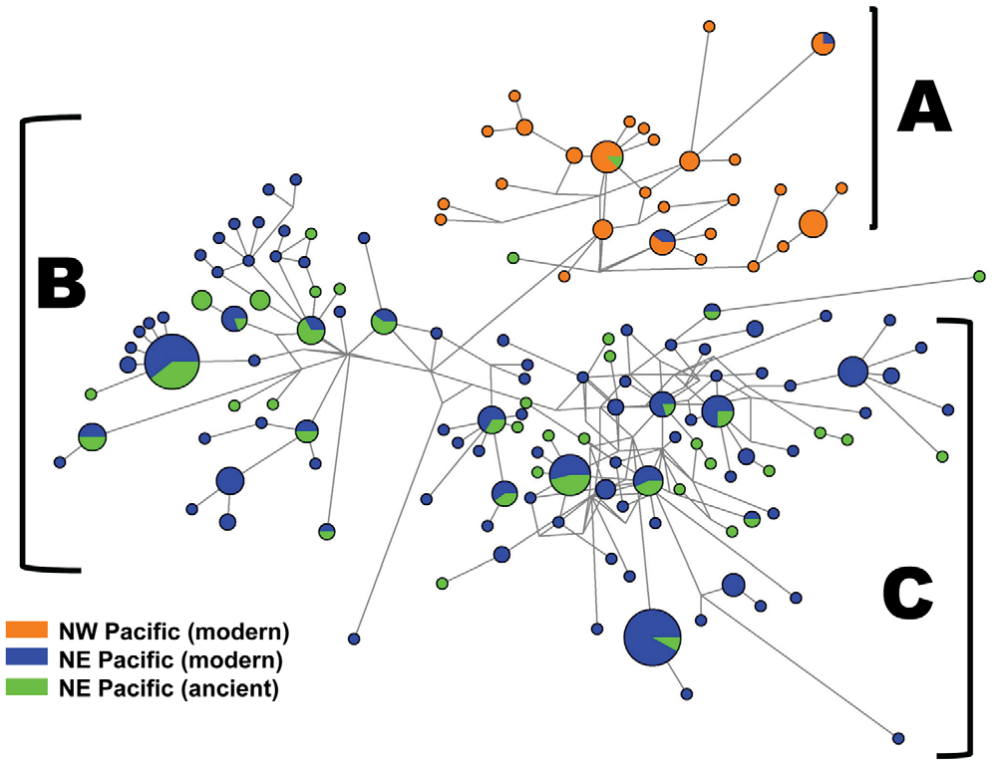
Median-joining network displaying ancient and modern D-loop haplotypes. Median-joining network displaying the relationships between the ancient D-loop haplotypes and modern herring populations from different geographical regions (modern datasets are those listed in Table S2 from [19]). The majority of the ancient samples cluster with modern herring in the two lineages from the northeast Pacific (lineages B and C). Lineage A is distinguished by 3 polymorphisms (15682C, 15841C, 15838G, coordinates based on Genbank accession NC009578); Lineages B and C are distinguished by three polymorphisms: lineage B resembles NC009578, while lineage C is defined by 15670C, 15823G, 15831T.

The minimum spanning network (Fig. 3) describing the phylogenetic relationships between the ancient and modern D-loop sequences demonstrates the presence of three distinct haplogroups: haplogroup A predominantly encompassing the populations of Western Pacific herring (Russia and Bering Sea), and haplogroups B and C predominantly encompassing samples from the Northeastern Pacific (southeast Alaska and British Columbia). With the exception of samples CP49 from southeast Alaska, and CP68 from Barkley Sound, all the ancient British Columbia and Alaska samples clustered into these two Northeastern Pacific lineages.

**Figure 4:**
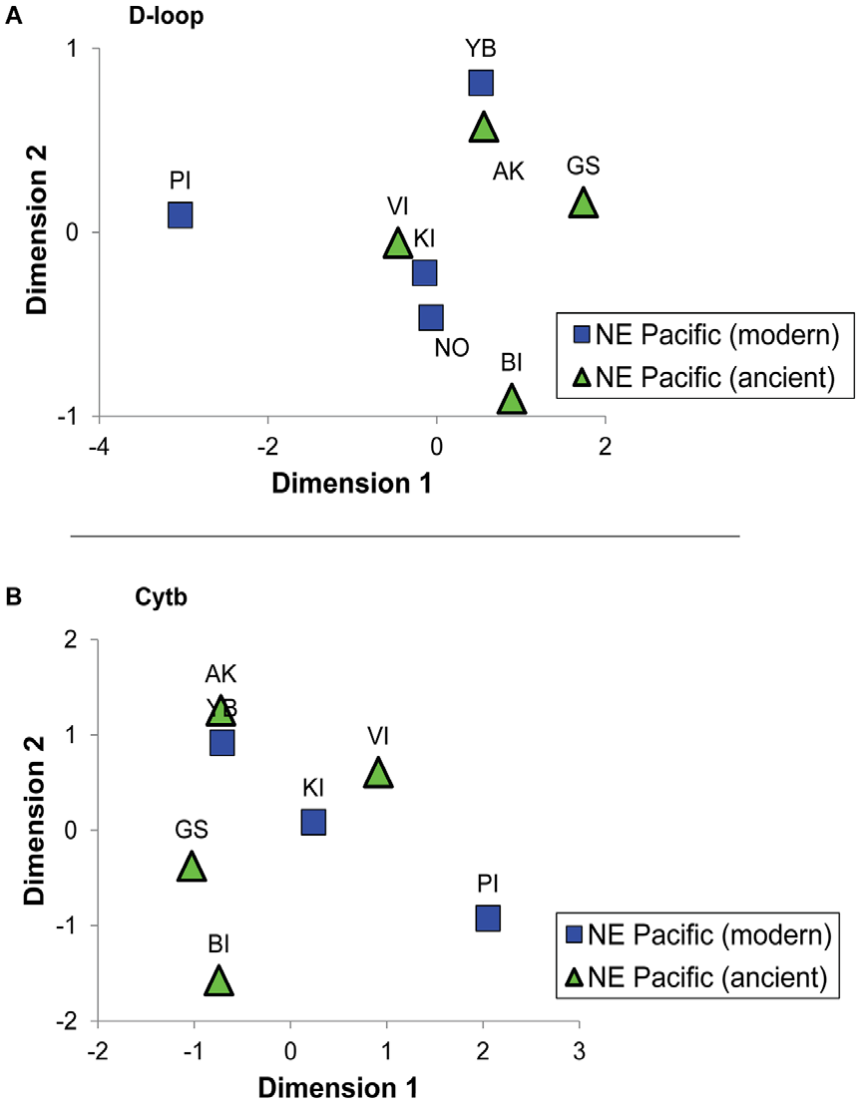
Multidimensional scaling plot of *Φ_ST_* between samples. A) D-loop (*r^2^* = 0.999) and B) cytb (*r^2^* = 0.985). YB is Yakutat Bay (southeast Alaska), KI is Knight Inlet (northern Georgia Strait), PI is Portage Inlet (southeastern Georgia Strait), NO is Nootka Sound (west coast of Vancouver Island), AK is Southeast Alaska, BI is Burrard Inlet, GS is northern Georgia Strait, VI is West Coast of Vancouver Island.

Ancient herring individuals from all four geographic regions (i.e. southeast Alaska, Georgia Strait, Burrard Inlet, west coast of Vancouver Island) are generally equally distributed between haplogroups B and C, with few shared haplotypes between sites. Hierarchical AMOVAs comparing archaeological herring samples with modern DNA data showed little spatial differentiation and no temporal differences (Table 1). Most differentiation was due to a modern sample collected from Portage Inlet (Puget Sound, southern Georgia Strait), whose genetic distinctiveness may be due to rare stochastic events in this population [19]. Exclusion of that sample removed all significant differentiation. There was no difference between modern and ancient samples regardless of the inclusion of Portage Inlet (Table 1). Correspondingly, a multidimensional scaling (MDS) plot based on *Φ_ST_* between samples clustered archeological samples with modern samples and showed Portage Inlet as the only outlier (Fig. 4).

**Table 1:** Results of a hierarchical AMOVA.

	Cyt b		D-loop	
	Incl PI	Excl PI	Incl PI	Excl PI
**Among populations** **(** ***F_ST_*** **)**	0.011	−0.009	0.047***	−0.001
**Among populations** **within groups** **(** ***F_SC_*** **)**	0.024*	0.001	0.048***	0.002
**Between groups (** ***F_CT_*** **)**	−0.013	−0.010	0	−0.003

Results of hierarchical AMOVA on each mtDNA region, with ancient and modern samples as the two groups. Modern samples [19] included were Portage Inlet, Knight Inlet, Yakutat Bay for cytb, and Portage Inlet, Knight Inlet, Nootka Sound and Yakutat Bay for D-loop. Portage Inlet (PI) was an outlier for all analyses, so AMOVAs are presented with and without that sample.

### Microsatellites

Three microsatellite loci were amplified in a subset of 22 archaeological herring remains (Table S4); all but one sample successfully amplified at least one nuclear locus. Nuclear DNA amplification success rates were inversely correlated with the length of the targeted allele, with the highest amplification success rate (21/22 samples) observed with CP4b (97–108 bp) and the lowest success rate (16/22 samples) observed with CPA 103c (179–247 bp). Rerunning all 22 samples at one microsatellite locus (Cha113) revealed three allele drop-out errors involving the three largest alleles, and two genotypes with mis-scoring due to stuttering, resulting in an overall error rate of 22.7%. There was no statistical evidence for large allele drop-out within loci or scoring errors due to stuttering in the ancient samples. Nevertheless, tests for Hardy-Weinberg equilibrium revealed significant heterozygote deficits at all three loci, with relatively high *F_IS_* values (Cha113: *F_IS_* = 0.215, Cpa103: *F_IS_* = 0.540, Cpa4: *F_IS_* = 0212). Correspondingly, expected heterozygosity was comparable to two modern herring samples from Puget Sound (Cherry Point and Quartermaster Harbor) [21] (*H_e_* = 0.865 vs *H_e_* = 0.907), but observed heterozygosity was lower (*H_o_* = 0.598 vs. *H_o_* = 0.898). There was significant genetic differentiation between the ancient sample and the two modern samples (to Quartermaster Harbor: *F_ST_* = 0.016, P = 0.057; to Cherry Point: *F_ST_* = 0.038, P = 0.001), but not between the two modern samples (*F_ST_* = 0, P = 0.676) Altogether, these microsatellite trials, although preliminary, suggested high error rates that could affect the power of analyses and may produce artificial differentiation between samples.

### SNPs

One SNP assay was tested on a subset of 30 modern and 30 ancient herring samples, and successfully genotyped 28 and 22 of the modern and ancient samples, respectively. Repeat genotyping revealed no discrepancy in the modern samples, but four out of 22 genotypes differed in the ancient DNA samples (Table S5). All errors were due to individuals scored as heterozygotes in one run and homozygotes in another, and in three of these discrepancies, a third run confirmed the genotypes as heterozygotes (the fourth sample did not amplify). Counting the two repeat runs in ancient samples, the error rate per PCR reaction for this SNP locus was therefore 4.5%. The higher number of undetermined genotypes in the ancient herring samples was due to poor amplification or to fluorescence scores intermediate between two established genotypes (Fig. S3). Three of the samples which displayed SNP genotyping errors (CP32, CP33, CP34) also displayed large allele drop-out or PCR failure in the microsatellites (the fourth sample, CP2, was not included in the microsatellite analysis).

In general, the samples displayed an inverse correlation between nuclear DNA preservation and qPCR cycle threshold (Ct). The modern samples displayed an average Ct value of 25.7, while the Ct for the ancient SNP samples (averaged over three runs) ranged between 32.7 and 47.7, with an overall average of 36.8. More than half the ancient samples (58.3%) with above average Ct values displayed errors or undetermined SNP genotypes, compared with only 17.6% of samples with lower than average Ct values. All four ancient samples with SNP genotyping errors had Ct values greater than 37.0. Tests for deviation from Hardy-Weinberg equilibrium were not significant in both the ancient and the modern sample. Heterozygosity was marginally higher in the modern sample (*H_e_* = 0.50) than in the ancient herring (*H_e_* = 0.44), but there was no significant differentiation between ancient and modern samples (*F_ST_* = 0.011, *P* = 0.226).

### Power Analysis

**Figure 5:**
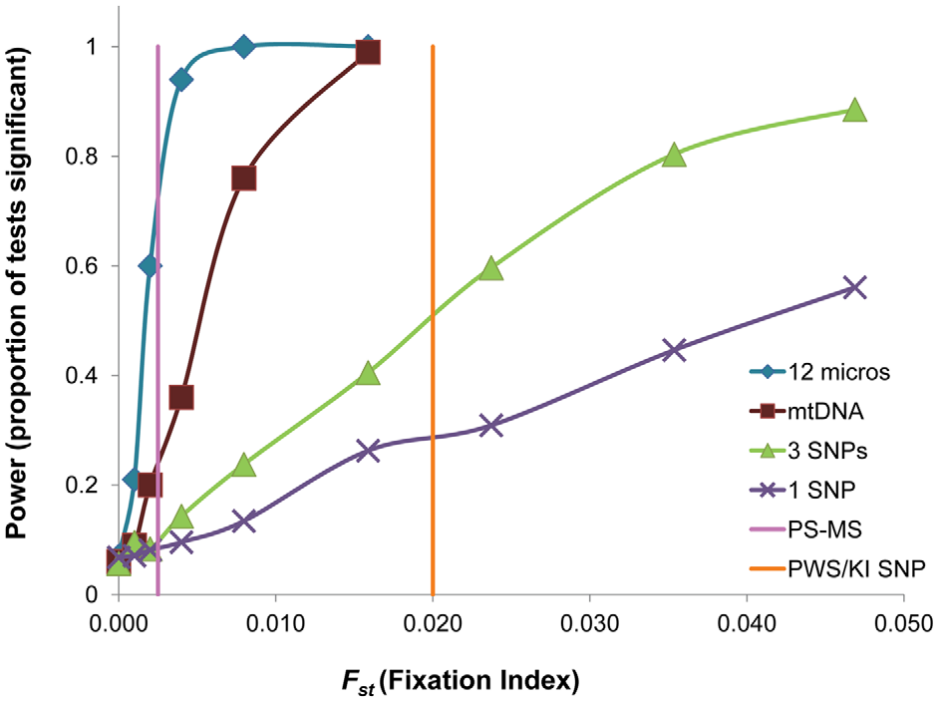
Power to detect significant genetic differentiation between two populations with a sample size of 48 individuals each with different marker sets. We compared data from 12 empirical microsatellites [21], empirical mtDNA haplotypes from the present study and three and one simulated biallelic SNP loci (3 SNPs, 1 SNP) with even allele frequencies. For comparison, the empirical microsatellite *F_ST_* values found in Puget Sound herring (PS-MS, [21]) and the empirical *F_ST_* of an outlier SNP found between Prince William Sound and Kodiak Island (PWS/KI SNP, [64]) are shown.

Power analyses comparing the three marker systems confirmed that approaches employing more markers with higher variability were more powerful in detecting significant population differentiation at a given *F_ST_* (Fig. 5). Data from 12 empirical microsatellites with high average heterozygosity (*H_e_* = 0.870) were most powerful, followed closely by the single locus mtDNA (*h* = 0.956). Only microsatellites had a reasonable chance to detect differentiation as low as observed between populations in Puget Sound (*F_ST_* = 0.0025). Because of their biallelic nature (max *H_e_* = 0.5), SNPs had lower power, though three SNPs had almost 90% power to detect population differentiation if the true *F_ST_* was as high as 0.05. Higher differentiation, as commonly found in genes under selection, resulted in greater power: for example, a single SNP locus with an *F_ST_* of 0.35 resulted in 73% power, and three SNP loci with *F_ST_* = 0.2 provided 92% power to detect significant genetic differentiation.

## Discussion

In this study, we sought to establish the authenticity and accuracy of genetic data from DNA extracted from archaeological skeletal remains of Pacific herring. Our expectation was that amplification success may be lower than in modern samples, but that resulting data would not be sufficiently biased to compromise their applicability for large scale studies on temporal aspects of the population dynamics of herring. Overall, the results point to excellent mitochondrial and nuclear DNA preservation in archaeological herring bone samples recovered from archaeological sites in the Northeast Pacific. Mitochondrial haplotype diversity was high in the ancient remains, comparable with that found in contemporary populations. Amplification of microsatellites was successful, but preliminary analyses revealed relatively high error rates and some evidence for large allele drop-out or stuttering in the ancient samples. In contrast, error rates at the tested SNP was relatively low, with no evidence for deviations from Hardy-Weinberg equilibrium. Taken together, our results support the accuracy of mtDNA and SNP data from ancient herring remains, taking into account the potential errors that may be caused by DNA degradation. Here, we discuss the implication of our results, discuss potential errors and their prevention in larger scale studies, and consider the goal of reconstructing ancient herring population structure from genetic analysis of archaeological remains.

### Authenticity of the aDNA Sequences

Due to the generally low quality and quantity of DNA template in ancient remains, ancient DNA analysis is particularly vulnerable to contamination from modern sources [50], [51]. The microsatellite, SNP, and mtDNA results obtained in this study can be attributed to authentic ancient herring DNA rather than contamination from modern sources. Several lines of evidence can be used to secure the authenticity of the ancient DNA sequences, including: (i) the use of dedicated ancient DNA facilities equipped with UV filtered ventilation, positive airflow, and bench UV lights; (ii) the separation of pre- and post-PCR workspaces; (iii) a vigorous chemical decontamination protocol of the bone samples prior to DNA extraction; and (iv) the absence of amplification in all of the of blank extracts and PCR negative controls. Multiple mtDNA haplotypes were identified within the samples as a whole as well as within each extraction batch, suggesting a lack of cross-sample contamination. The ancient remains demonstrated appropriate molecular behavior, as: a) higher amplification success rates were observed in mtDNA compared to nuclear DNA, which is expected based on the higher quantity of mtDNA per cell; and b) shorter microsatellite amplicons amplified more readily than longer amplicons. While repeat extractions from the same herring bone were precluded by the small size of the samples, repeat amplifications from the same bone DNA extracts consistently yielded the same mtDNA sequence. Additionally, contamination from modern herring DNA can generally be excluded since modern herring samples were extracted and analyzed in a separate laboratory, and only after analysis on the ancient samples had been completed.

### Ancient DNA Preservation

The first objective of this study was to determine whether mitochondrial and nuclear DNA could be obtained from archaeological herring bones. At least one region of mtDNA was amplified from 91% of the archaeological samples, demonstrating the excellent mtDNA preservation in archaeological herring bones. This success rate is consistent with other ancient DNA studies of both fish and mammal remains on the Pacific Northwest which demonstrate success rates ranging from 73–97% for mtDNA extraction and amplification [39], [40], [52]–[54] (see Grier et al. [41] for an exception). Of the eight failed samples, six were recovered from the Kahkeeky archaeological site, suggesting that specific taphonomic factors at that site play an important role in overall DNA preservation. Nuclear DNA, either as SNPs or microsatellites, were amplified from 72–95% of the tested samples (all of which had yielded well preserved mtDNA). Very low frequencies of damage derived or polymerase enzyme modifications were observed in the ancient mitochondrial DNA sequences. Moreover, up to 290 bp fragments of mtDNA, and up to 147 bp fragments of nuclear DNA were routinely amplified from extremely small bone samples, often weighing less than 10 mg. Considering that ancient DNA studies typically target bone samples ranging from 0.2–1.0 g, the fact that authentic, replicable mtDNA sequences as well as nuclear microsatellites and SNPs could be obtained from as little as 3 mg of bone pushes the limits of ancient DNA research. DNA was successfully retrieved from samples ranging from 100YBP to 3800 cal BP. No differences in amplification success rate were observed between samples dating to different millennia; instead, DNA preservation seemed to be more closely associated with depositional context, with poor DNA preservation observed only at the Kahkeeky site. These observations, in combination with previous ancient DNA studies on fish and mammal remains from the Northwest Coast of North America, indicate that this region provides extremely favorable conditions for DNA preservation [39], and enhances the feasibility for including ancient DNA data into modern environmental management studies.

The qPCR SNP approach also yielded useful information concerning overall DNA preservation. Samples with relatively higher Ct values demonstrated more errors and undetermined genotypes than those with lower Ct values. Samples which displayed large allele drop-out in the microsatellites also displayed higher than average Ct values in the corresponding SNP assay. These results suggest that qPCR Ct values can act as a proxy for overall DNA preservation [55]–[57], and could be used to select the best preserved samples for additional nuclear DNA analyses, and reduce the likelihood for genotyping errors and false positive results.

The use of archaeological bone samples, rather than complete skeletons, may raise concerns whether disarticulated bones can be attributed to individual fish, especially when sampling repetitive skeletal elements such as vertebrae. In this study, however, the possibility of sampling multiple bones from the same individual is minimal as many amplified samples were recovered from spatially and temporally distinct archaeological contexts or layers (Table S1). Moreover, most samples from the same context revealed different mtDNA sequences. More intensive sampling of layers with tight chronological control would produce a specific temporal ‘snapshot’ of the population, while application of more nuclear loci would allow identification of bones from the same individual. Nevertheless, the collection of representative samples from short time periods without duplicated individuals will remain an important consideration in ancient DNA studies of herring.

### Comparison of Molecular Markers

Although both the cytb and the D-loop sequence from ancient samples were necessarily shorter than those used in modern studies (see methods), they showed similar patterns of genetic diversity. Diversity was much lower at cytb than at D-loop (Fig. 2), and the three clades described by Liu et al. [19] were less obvious, other than in large differences in genetic diversity between western and eastern Pacific herring populations (Fig. S2). In the D-loop data, however, the ancient samples clearly fell within the three haplogroups. Nevertheless, no genetic differentiation was found among the four geographic regions represented by the archaeological samples, ranging from southeast Alaska to southern Vancouver Island. This lack of differentiation is similar to that described for modern samples [19], [20], with the only outlier represented by a late spawning population in Portage Inlet (also detected in this study using shorter D-loop sequences). Our results therefore suggest that at least with D-loop, the use of shorter sequences commonly employed for ancient DNA analyses is sufficient and comparable to results with longer sequences available with modern DNA. Such data would be extremely useful to investigate long-term temporal changes in broad scale genetic patterns using coalescence approaches [20], [58], [59], although the resolution of such analyses may be insufficient to investigate the effects of industrial fishing in British Columbia over the past century. Indeed, the lack of differentiation between ancient and modern herring despite relatively large sample sizes supports the notion that reproducible data were obtained from representative samples. Nevertheless, it also raises concerns about the power of mtDNA for the analysis of small scale population structure in high gene flow species such as herring, which has implications for fisheries management.

Past studies [42], [60] as well as our power analyses suggest low discriminatory power of mtDNA, which is inherited as a single locus and therefore provides less discriminatory power than multilocus nuclear DNA analyses. For example, 12 microsatellite loci of similar variability as mtDNA would have 80% power to detect an *F_ST_* = 0.003 (detected among Puget Sound herring populations [21]), while the corresponding power of mtDNA would be less than 20%. Yet microsatellites proved to be problematic in our study; there were significant deficiencies of heterozygotes, apparently resulting from large allele drop-out and stuttering issues. Because of the inherent size differences between microsatellite alleles, this marker system may be particularly prone to preferential PCR amplification of specific alleles, especially in degraded DNA consisting of short fragments [61]. In this study, shorter microsatellites amplified more readily than longer ones, indicating that a greater success could be obtained by using high-throughput sequencing to discover shorter loci (<100 bp) [61].

A multitube PCR approach can also be used to more confidently discriminate between homozygotes and heterozygotes. Taberlet et al. [62] recommend at least seven independent PCR reactions in order to type homozygotes with a confidence level of 99%. In their microsatellite study of fossil moas, Allentoft et al. [61] accepted homozygote profiles after four unambiguous independent simplex PCR reactions, and heterozygote profiles if they demonstrated unambiguous bi-allelic profiles, and/or two different unambiguous mono-allelic profiles in different PCRs. Even using the more practical approach of Allentoft et al., well preserved samples require at least three to four independent PCR reactions for each microsatellite, with more deteriorated samples requiring as many as six to 10 PCRs. Though the multitube approach can provide robust microsatellite data with high confidence, the amount of DNA template required limits the number of loci that can be tested, especially in poorly preserved samples which require more than four independent PCRs. Unlike larger mammal or bird bones, the small size of the herring bones precludes the opportunity for obtaining additional DNA template from a repeat extraction. It therefore appears unlikely that a large-scale population survey based on archaeological material would be feasible using multiple microsatellites.

Because of their biallelic nature, SNPs have less power per locus than either microsatellites or mtDNA (Fig. 5). However, in contrast to mtDNA and most microsatellites (see [63] for a notable exception), some SNPs show much higher differentiation than predicted from neutral expectations because they are located within, or close to, genes under selection. For example, SNPs with an *F_ST_* of 0.02 were detected in a preliminary survey of herring in Prince William Sound and Kodiak Island in Alaska [64], and a recent survey of genetic variation in Atlantic herring revealed SNPs with *F_ST_* values as high as 0.35 [65] on geographic scales comparable to the present study. Very few of such high-differentiation loci would provide high power to detect genetic differentiation, and would also allow a multi-tube approach to avoid genotyping errors. Such SNPs would have to be discovered using large scale genome scan approaches, but such methods have already been successfully applied in both Atlantic [66] and Pacific herring [64] and are continually becoming faster, cheaper and more efficient [67].

Compared with modern samples, the ancient herring bones displayed higher frequencies of failed SNP amplifications, undetermined genotypes, and higher error rates, likely due to the lower quantity and quality DNA template, as well as stochastic effects [68], [69]. Using a multiple tube and consensus genotyping approach, replicable SNP results were obtained for over 70% of the ancient samples, with a relatively low error rate of 4.5% per reaction. Allele drop-out, however, continues to be an issue for nuclear DNA analysis, as demonstrated by the higher proportion of homozygotes in the ancient herring compared to modern samples [68]. The optimal DNA input for SNPs ranges from 1–10 ng per reaction, with successful amplification using as little as 40 templates/reaction [69]. Our study further supports this observation, indicating that SNPs represent the optimal method for population-level genetic analyses of ancient herring. Compared with microsatellites, SNP data can be more easily standardized between laboratories, allowing for population comparisons across large regions and multiple researchers. Using the qPCR approach, SNPs are also more amenable to high-throughput genotyping, especially once assays have been optimized for low quantity template (i.e., targeting short DNA templates with highly sensitive PCR amplification protocols).

### Potential for aDNA Analyses in Applications of Modern Fisheries Management

The results of this study demonstrate a high potential for incorporating ancient DNA analyses into modern fisheries management by extending the temporal scale of observation critical for assessing the degree of change from the pre-industrial era. Understanding herring spatial diversity is fundamental for advancing fisheries management, and the ability to discern herring population structure is highly dependent on the time scale of observation. This study indicates that archaeofaunal remains provide excellent source material for characterizing more detailed patterns of Pacific herring genetic diversity through time and space. Herring remains are abundant in Eastern Pacific archaeological sites ranging from Alaska [10] to Baja California [70], often occurring in regions where herring no longer spawn. Given the depleted state of herring stocks today, ancient DNA analysis of archaeological data may now represent the only way to assess former genetic diversity and abundance, and explicitly test hypotheses concerning the genetic differentiation of herring populations in the past.

Current herring management in British Columbia is based on relatively recent observations of a high degree of exchange between spawning grounds, and the presence of few distinct, reproductively-isolated populations. Recent research, however, has indicated that locally adapted populations may be most vulnerable to anthropogenic influences, with population level extinction rates often occurring at orders of magnitude higher than species level extinctions [71]. For example, it is estimated that 29% of nearly 1400 historically documented Pacific salmon populations have already been extirpated in the Pacific Northwest [72]. This study provides a robust technical approach for identifying additional, extirpated herring populations through ancient DNA analysis which has significance for current herring management practices based in part on genetic characterization of modern populations. Not only may the loss of diversity affect the ability of herring to adapt to external forces and to rebuild from low population levels [73], but it may also necessitate the redefinition of current herring management units.

This study provides the necessary foundation for wider scale studies on temporal genetic variation in herring. Traditional ecological knowledge, archaeological data, and historical records can lead to hypotheses concerning past herring genetic diversity that can be explicitly tested using ancient DNA data, including assessing past levels of between-region genetic differentiation, the biological relationship between 'resident' and migratory stocks within regions, and the identification of locally-adapted distinct herring populations. Our study suggests that SNP analyses of ancient remains provide the best methodological approach for characterizing genetic diversity, and identifying distinct populations in the past. This interdisciplinary approach has the potential to enhance not only fisheries management, but investigations into the wider effects of industrial fishing practices, climate change, First Nations traditional herring management practices and Aboriginal rights and titles.

## Materials and Methods

### Ethics Statement

All necessary permits were obtained for archaeological excavations. For Alaska sites (analysed by MM), the Tongass National Forest and Craig Ranger District granted permission for excavations at Cape Addington (49-CRG-188) and Coffman Cove (49-PET-067; AG-0109-C-0053). The State of Alaska, Office of History and Archaeology, granted permission for excavation at the Coffman Cove Ferry Terminal Site (49-PET-556; Project 67844). The Craig Community Association, Klawock Cooperative Association, Hydaburg Cooperative Association, and the Wrangell Cooperative Association also supported these projects. For Northern Georgia Strait, permits for excavations in Desolation Sound (Sliammon/Grace Harbour) (awarded to DL), including permissions from Sliammon First Nation, Desolation Sound Marine Park (Permit #103536), and the British Columbia Provincial Archaeology Branch (Permit 2008-0299). For Burrard Inlet (awarded to DL), permissions were obtained from Tsiel-Watuth First Nation, the Municipal Parks Authorities (North Vancouver District, Greater Vancouver Regional District Parks Permit), the British Columbia Provincial Archaeology Branch (Permit #LM08116359), and Heritage Conservation Act (Permit 2008-0172). For Barkley Sound, permission was obtained from the Parks Canada Agency under Research and Collection Permits: #PRN-2008-1579 and #PRN-2009-2737 for the Broken Group Islands unit of Pacific Rim National Park Reserve (Ucluelet, BC).

No analyses in this study were conducted on living fish. This research included ancient DNA analysis of herring bones recovered from archaeological contexts hundreds to thousands of years old. Genetic analysis was also conducted on naturally deposited herring roe, non-invasively collected from kelp or other substrates during spawning season.

### Archaeological Sites

Ancient DNA analysis was applied to 85 archaeological herring samples recovered from four regions in Alaska and British Columbia (Table S1, Fig. 1). The majority of the samples were herring vertebrae, often weighing less than 10 mg (Fig. S1).

### DNA Extraction

The herring bone sample preparation and DNA extraction were conducted in the dedicated Ancient DNA laboratory at Simon Fraser University (SFU) following strict protocols for contamination control and detection [52], [74]. DNA was extracted following a modified silica spin column protocol [75]. Bone samples were chemically decontaminated to remove possible surface contamination: bone samples were immersed in 100% commercial bleach (6% sodium hypochlorite) for 7 min, and rinsed twice in distilled water. Bone samples were then UV irradiated for 30 min on two sides. Following decontamination, DNA was extracted following the procedure described in Yang et al. [76].

Thirty modern samples of *C*. *pallasi* roe were also analyzed in this study to validate the SNP assay. The modern samples were collected from spawning aggregations on the Central Coast of British Columbia in 2011 and stored in 70% ethanol at 4°C. Sampled sites included Idle Point, Kwakume Inlet, Powell Anchorage, Spiller Channel and Troup Passage (Fig. 1). A single egg from each roe sample was isolated for DNA extraction using a DNeasy Blood and Tissue Kit (Qiagen, Valencia, CA) according to manufacturer’s protocols. Modern herring samples were processed in a separate laboratory at SFU dedicated to modern DNA analysis.

### Mitochondrial DNA Analyses

PCR primers were designed to target two different fragments of the *Clupea* mitochondrial genome (Table S6), including a 298 bp fragment of the cytb gene, and a 418 bp fragment of the D-loop (amplified in two overlapping fragments of 283 bp and 295bp, respectively). PCR amplifications were conducted in a Mastercycler® ep (Eppendorf) in a 30- or 50-µL reaction volume containing 50 mM KCl, 10 mM Tris-HCl, 2.5 mM MgCl_2_, 0.2 mM dNTP, 1.0 mg/mL BSA, 0.3 µM each primer, 3.0–5.0 µL DNA sample and 2.5–3.5 U AmpliTaq Gold LD (Life Technologies Corporation, Carlsbad, CA). PCR began with an initial 12-min denaturing period at 95°C, followed by 60 cycles at 94°C for 30 s (denaturing), 52°C for 30 s (annealing), and 72°C extension for 40 s. Blank extracts and negative controls were included in each of the PCR sets. PCR products were sequenced using both forward and reverse primers at Eurofins MWG Operon, Inc., Huntsville, Alabama. A subset of the samples underwent replication of PCR and sequencing to check the reproducibility of the results and to detect any base pair misincorporations due to DNA damage. Repeat amplification and sequencing of both D-loop fragments took place for 23 samples, with repeat amplification and sequencing of one D-loop fragment for an additional 12 samples. Nine samples underwent repeat amplification and sequencing of the cytb fragment.

The ancient herring sequences were visually edited and truncated to remove primer sequences using ChromasPro software (www.technelysium.com.au). The D-loop consensus sequences were truncated to 350 bp once primer sequences were removed. Multiple alignments of the ancient sequences and available Genbank reference sequence were conducted using ClustalW [77] and BioEdit (www.mbio.ncsu.edu). The ancient cytb sequences were compared to modern published references through the GenBank BLAST application (http://www.ncbi.nlm.nih.gov/BLAST/), and compared to Pacific (n = 46) and Atlantic (n = 13) herring sequences obtained from GenBank to ensure accurate species identification. Species identifications were assigned only if a sequence matches identically or very closely with published reference sequences, and if no other evidence, including reproducibility tests or additional sequencing of the same sample (i.e. D-loop sequences) indicated a different species. The 74 ancient D-loop sequences were phylogenetically compared to 234 sequences downloaded from GenBank, including sequences from Western (n = 50) and Eastern (n = 184) Pacific herring [19]. Reference sequences were truncated to include only the 350 bp of the mitochondrial D-loop targeted in the ancient samples.

Bayesian (Monte Carlo–Markov chain) consensus trees analyses were conducted using MrBayes 3.1.2 [78] with the model parameters (K80+G) identified through MrModelTest 2.3 [79]. Cytb consensus trees were generated from two runs of MrBayes using 10 million generations each. Median-joining networks of the ancient D-loop haplotypes and select Pacific herring reference sequences were produced through Network (v. 4.5.1.6) [80]. Haplotype (*h*) and nucleotide diversity values (π) for each archaeological site, as well as for modern datasets [19] were calculated with Dna-SP v.5.10.01 software [81]. Pairwise *Φ_ST_* and AMOVAs were calculated in Arlequin v 3.5 [82], and *Φ_ST_* values were used in a MDS analysis procedure in SPSS v 11 (ALSCAL, SPSS Inc.) [83] to display genetic relationships between ancient and modern samples [19].

### Microsatellite Analysis

Twenty-two of archaeological herring remains that amplified successfully using the mtDNA primer sets were amplified using three separate microsatellite loci. CPA 103c targeted a 179–247 bp fragment [84]; CPA4b targeted 97–108 bp [85] and CPA113a targeted 100–150 bp [86]. At SFU, each microsatellite was amplified in a simplex reaction with the same composition and PCR conditions as the mtDNA amplifications. Microsatellite fragment sizes were determined in the School of Aquatic and Fishery Sciences at the University of Washington (UW) on a MegaBace 1000 using manufacturer’s software. Genotype frequencies were tested for evidence of stuttering and large allele drop-out in Microchecker [87] and for conformance to Hardy Weinberg equilibrium by exact tests in GenePop v4 [88]. GenePop was also used to test for significance of genic population differentiation and to calculate *F_ST_* values according to Weir & Cockerham [89].

### SNP Analyses

We developed one SNP assay from Roberts et al. [64] to test the potential for amplifying single copy nuclear DNA from the ancient herring remains. The 114bp TaqMan® custom assay Cpa_11961_c04 (Life Technologies Corporation, Carlsbad, CA) was designed using Primer Express software (Table S7). SNP genotyping was conducted at SFU using an Applied Biosystems StepOne™ Real-Time PCR instrument (Carlsbad, CA) in a 15 µL reaction volume containing 1.5× GeneAmp® Gold Buffer (Life Technologies Corporation, Carlsbad, CA), 2.5 mM MgCl_2_, 0.2 mM dNTP, 1.0 mg/mL BSA, 500 nM of ROX™ Passive Reference Dye (Affymetrix, Santa Clara, CA), 1× TaqMan® assay™ (Life Technologies Corporation, Carlsbad, CA), 2 µL of DNA extract and either 1.5 U (modern samples) or 2.25 U (ancient samples) of AmpliTaq Gold™ regular or AmpliTaq Gold™ LD (Life Technologies Corporation, Carlsbad, CA). PCR amplifications had a pre-read stage (60°C for 30 s), an initial denaturation step (95°C for 12 min) follow by 60 cycles of a denaturation step (95°C for 15 s) followed by an annealing/extension step (60°C for 90 s) and finally a post-read stage (60°C for 30 s).

Thirty ancient and 30 modern herring were tested using the SNP assay. All PCR reagents and reactions were prepared in the ancient DNA laboratory at SFU; ancient herring DNA extracts were added in the ancient DNA laboratory, while modern herring DNA extracts were added in the modern DNA laboratory at SFU. All PCR amplifications were conducted in a separate post-PCR laboratory at SFU. Multiple no-template controls (NTC) were included in each PCR batch. Modern herring extracts with known genotypes were used as positive controls (undiluted when amplified with only modern samples and at 1∶50 dilution for when amplified with ancient samples) for all genotyping batches. Allele calls were made using the autocaller function of the StepOne™ Software v2.0 (Life Technologies Corporation, Carlsbad, CA) with the quality value set to 95%. For the 30 modern herring, a consensus genotype was declared if the same genotype was assigned to the sample twice out of duplicate runs. For the 30 ancient herring, a consensus genotype was declared for samples if the same genotype was assigned twice out of triplicate runs. We determined the error rate per reaction by dividing the number of allelic mismatches by the total number of PCRs [68] for one of the two or three batches. Tests for Hardy-Weinberg equilibrium and population differentiation between modern and ancient samples followed procedures outlined for microsatellites.

### Power Analysis

We evaluated the power of different marker systems by simulating populations in PowSim [90]. Briefly, this computer program simulated a single population with empirical or assumed allele frequencies that split up into two subpopulations of 1000 individuals each, which were sampled (n = 48) after a determined number of generations of drift. We compared allele and haplotype frequencies of the two samples using Fisher’s exact tests for microsatellites and *χ^2^* values for SNPs following recommendations in Ryman [91]. Each simulation consisted of 1000 repetitions. Initial allele frequencies for microsatellites are based on Mitchell [21] and for mtDNA from the ancient sample presented in this study; SNP loci had two alleles with a frequency of 0.5 each. The variance in *F_ST_* values between repeat runs of a specific simulation was much larger for SNPs than for microsatellites or mtDNA – instead of using all 1000 repeat runs of the simulation as for microsatellites and mtDNA, we therefore carried out 10,000 runs for SNPs and used only the simulations that were within ±10% of the expected *F_ST_* values. This approach was appropriate as we were interested to simulate the performance of SNPs under selection.

## Supporting Information

Figure S1 **Individual herring vertebra weighing less than 10 mg.**
(TIF)

Figure S2 **Phylogenetic tree of cytb haplotypes.** Bayesian (Monte Carlo–Markov chain) consensus tree displaying the relationships between the obtained cytb sequences and modern *Clupea* Genbank reference sequences (accession numbers listed), with Pacific anchovy (*Cetengraulis mysticetus*) as the outgroup. Posterior probabilities of the major nodes are listed for each of the branches. Clade CP1: Eastern Pacific Herring haplotypes; CP2: Western Pacific and Bering Sea Herring haplotypes; CH1: Atlantic herring (*C. harengus*).
(PDF)

Figure S3 **Example of Cpa_11961_c04 TaqMan SNP assay results.** Allelic discrimination plots for a) modern herring extracts, and b) ancient herring extracts. Samples called using the autocaller function of the StepOneTM Software v2.0 are indicated by either red (GG), green (GT) or blue (TT) circles. The squares indicate the NTC’s and the X’s indicate samples with either no amplification or undetermined genotypes.
(PDF)

Table S1 **Cytochrome** ***b*** **and D-loop haplotype of the analyzed archaeological herring bones.**
(DOCX)

Table S2 **Modern and ancient herring haplotype (** ***h*** **) and nucleotide (π) diversities** [**92**] **based on 350 bp mtDNA D-loop fragment.**
(DOCX)

Table S3 **Modern and ancient herring haplotype (** ***h*** **) and nucleotide (π) diversities** [**92**] **based on 235 bp mtDNA cytb fragment.**
(DOCX)

Table S4 **Microsatellite analyses.**
(DOCX)

Table S5 **Modern and ancient herring SNP genotypes.**
(DOCX)

Table S6 **Cytb and D-loop** ***Clupea*** **mtDNA PCR amplification primers.**
(DOCX)

Table S7 **Reporter sequence and primers for SNP assay.**
(DOCX)
