# Correction: High Potential for Using DNA from Ancient Herring Bones to Inform Modern Fisheries Management and Conservation

**DOI:** 10.1371/annotation/cd8623ff-b708-4972-8d59-803e56e77005

**Published:** 2013-06-25

**Authors:** Camilla F. Speller, Lorenz Hauser, Dana Lepofsky, Jason Moore, Antonia T. Rodrigues, Madonna L. Moss, Iain McKechnie, Dongya Y. Yang

The following funder in the Funding Disclosure is missing information: the Hakai Network for Coastal Peoples and Ecosystems.

The following is the correct funder: the Tula and Hakai Network for Coastal Peoples and Ecosystems. 

