# Correction: High Potential for Using DNA from Ancient Herring Bones to Inform Modern Fisheries Management and Conservation

**DOI:** 10.1371/annotation/1543dcf1-07b5-4630-9658-aac74cf85155

**Published:** 2013-07-19

**Authors:** Camilla F. Speller, Lorenz Hauser, Dana Lepofsky, Jason Moore, Antonia T. Rodrigues, Madonna L. Moss, Iain McKechnie, Dongya Y. Yang

This correction will replace the previous correction made by PLOS ONE. There is more information that is missing from the Funding Disclosure.

The first sentence in the Funding Statement should read:

This research was supported in part by a National Geographic Society Discovery grant (awarded to DYY and DL), a SSHRC Partnership grant (awarded to DL), the Tula Foundation and Hakai Network for Coastal Peoples and Ecosystems. 

