# Supplementary figures and images for: High Potential for Using DNA from Ancient Herring Bones to Inform Modern Fisheries Management and Conservation
Source: PLoS One. 2012 Nov 30;7(11):e51122. doi: 10.1371/journal.pone.0051122 (PMC3511397; doi:10.1371/journal.pone.0051122)

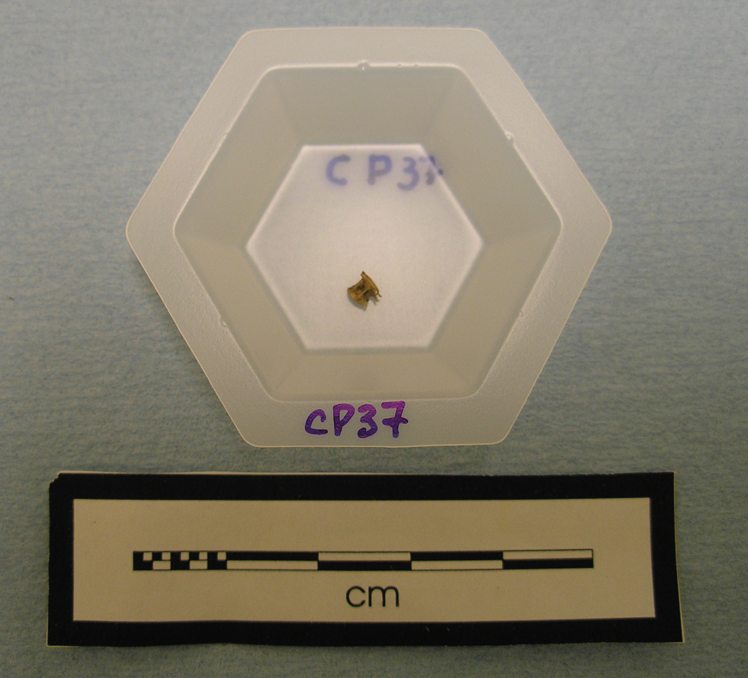

Supplement: Figure S1 — Individual herring vertebra weighing less than 10 mg. (TIF) [file pone.0051122.s001.tif]

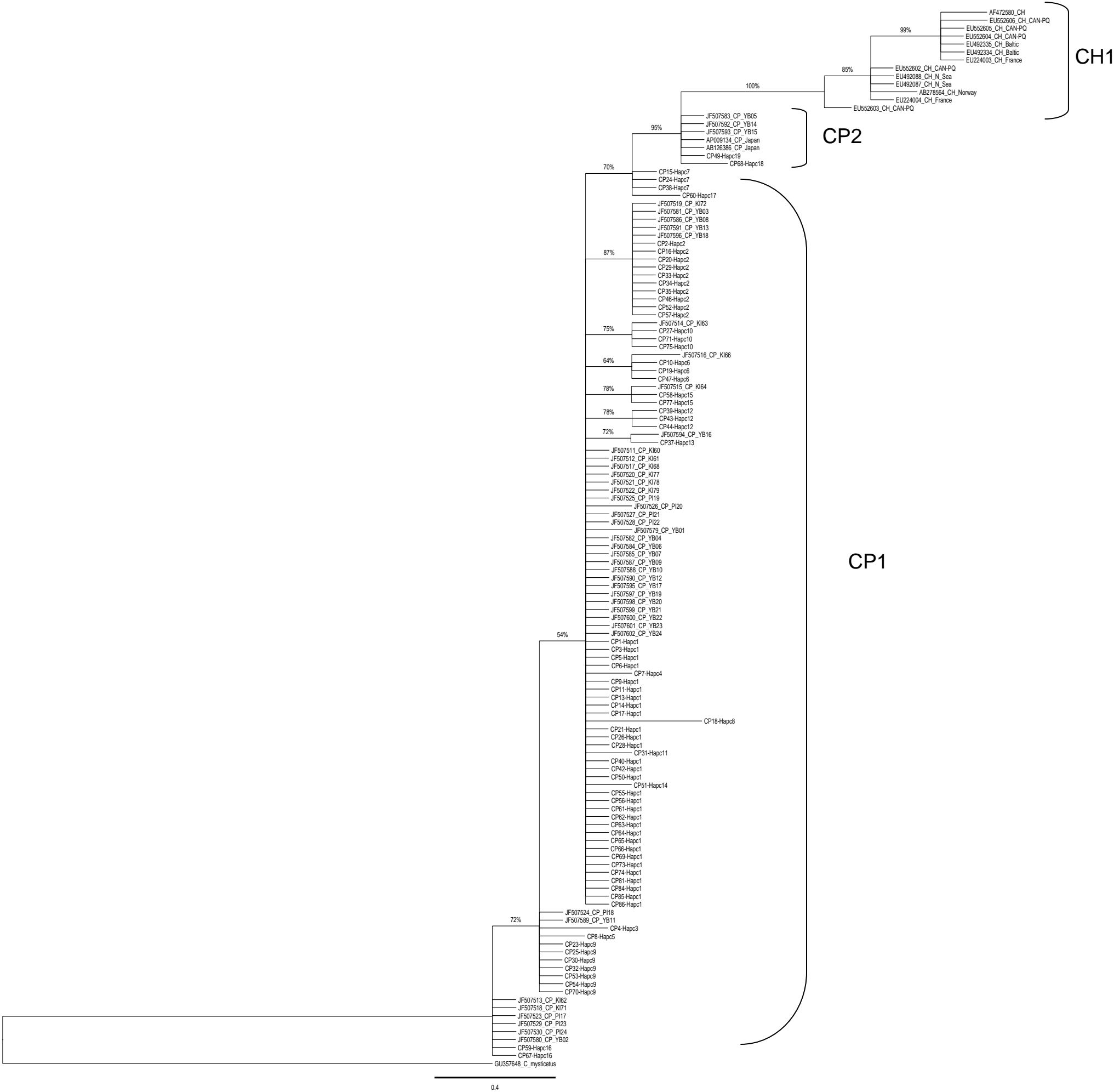

Supplement: Figure S2 — Phylogenetic tree of cytb haplotypes. Bayesian (Monte Carlo–Markov chain) consensus tree displaying the relationships between the obtained cytb sequences and modern Clupea Genbank reference sequences (accession numbers listed), with Pacific anchovy (Cetengraulis mysticetus) as the outgroup. Posterior probabilities of the major nodes are listed for each of the branches. Clade CP1: Eastern Pacific Herring haplotypes; CP2: Western Pacific and Bering Sea Herring haplotypes; CH1: Atlantic herring (C. harengus). (PDF) [file pone.0051122.s002.pdf]

Allele Discrimination Plot (SNP Assay: Cpa\_11961\_C04)

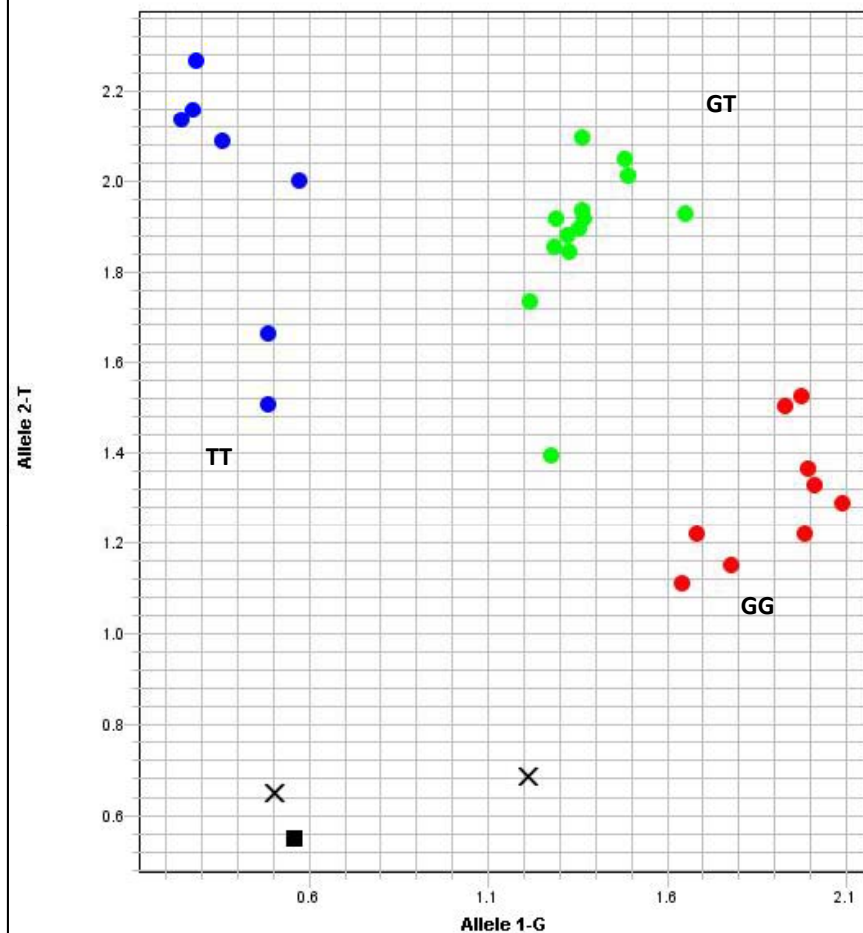

a

Allele Discrimination Plot (SNP Assay: Cpa\_11961\_Co4)

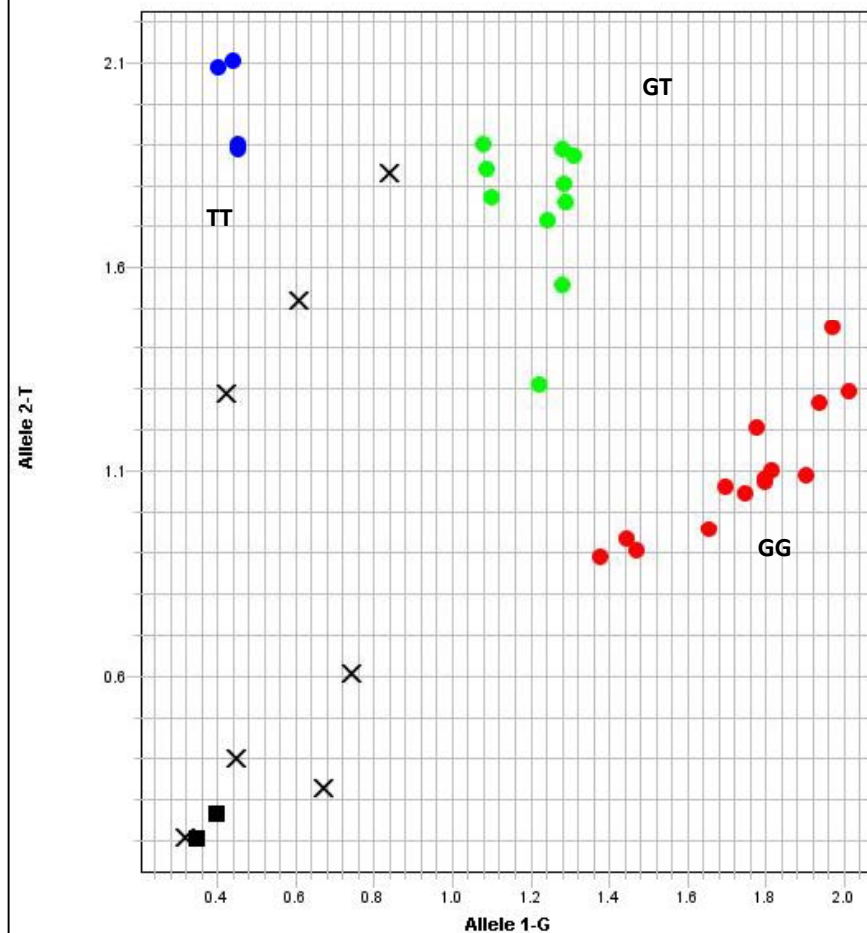

b

Supplement: Figure S3 — Example of Cpa_11961_c04 TaqMan SNP assay results. Allelic discrimination plots for a) modern herring extracts, and b) ancient herring extracts. Samples called using the autocaller function of the StepOneTM Software v2.0 are indicated by either red (GG), green (GT) or blue (TT) circles. The squares indicate the NTC’s and the X’s indicate samples with either no amplification or undetermined genotypes. (PDF) [file pone.0051122.s003.pdf]
