# Supplementary material for: High Potential for Using DNA from Ancient Herring Bones to Inform Modern Fisheries Management and Conservation
Source: PLoS One. 2012 Nov 30;7(11):e51122. doi: 10.1371/journal.pone.0051122 (PMC3511397; doi:10.1371/journal.pone.0051122)
Supplement: Table S1 — Cytochrome b and D-loop haplotype of the analyzed archaeological herring bones. (DOCX) [file pone.0051122.s004.docx]

**Table S1. Cytochrome *b* and D-loop haplotype of the analyzed archaeological herring bones.**

| **Lab Code** | **Element  (weight in g)** | **Archaeological Site** | **Location** | **Date (BP)** | **D-loop**  **haplotype** | **Cytb**  **haplotype** |
| --- | --- | --- | --- | --- | --- | --- |
| CP42 | Vertebra (<0.01) | Cape Addington Rockshelter | Southeast Alaska | 1340-1140cal BP | Hapd24 | Hapc1 |
| CP43 | Vertebra (0.01) | Cape Addington Rockshelter | Southeast Alaska | 1340-1140cal BP | Hapd25 | Hapc12 |
| CP44 | Vertebra (<0.01) | Coffman Cove Site | Southeast Alaska | 3800-3721 cal BP | Hapd26 | Hapc12 |
| CP45 | Vertebra (<0.01) | Coffman Cove Site | Southeast Alaska | 3800-3721 cal BP | Insufficient Data | Insufficient Data |
| CP46 | Vertebra (<0.01) | Coffman Cove Site | Southeast Alaska | 3800-3721 cal BP | Hapd12 | Hapc2 |
| CP47 | Vertebra (0.01) | Coffman Cove Site | Southeast Alaska | 3800-3721 cal BP | Hapd9 | Hapc6 |
| CP48 | Vertebra (<0.01) | Coffman Cove Site | Southeast Alaska | 3800-3721 cal BP | Hapd27 | Insufficient Data |
| CP49 | Vertebra (<0.01) | Coffman Cove Ferry Terminal | Southeast Alaska | 2330-2131 cal BP | Hapd28 | Hapc19 |
| CP50 | Prootics/pterotics (0.02) | Coffman Cove Ferry Terminal | Southeast Alaska | 2330-2131 cal BP | Hapd1 | Hapc1 |
| CP51 | Prootics/pterotics (0.01) | Coffman Cove Ferry Terminal | Southeast Alaska | 2330-2131 cal BP | Hapd29 | Hapc14 |
| CP01 | Unknown (0.02) | Klehkwahnnohm | Northern Georgia Strait | ~250BP | Hapd1 | Hapc1 |
| CP02 | Unknown (0.03) | Klehkwahnnohm | Northern Georgia Strait | ~250BP | Hapd2 | Hapc2 |
| CP03 | Vertebra (<0.01) | Klehkwahnnohm | Northern Georgia Strait | ~250BP | Hapd3 | Hapc1 |
| CP04 | Vertebra (0.02) | Klehkwahnnohm | Northern Georgia Strait | ~250BP | Hapd4 | Hapc3 |
| CP05 | Quadrate (<0.01) | Klehkwahnnohm | Northern Georgia Strait | ~250BP | Hapd3 | Hapc1 |
| CP06 | Vertebra (0.01) | Klehkwahnnohm | Northern Georgia Strait | ~250BP | Hapd5 | Hapc1 |
| CP07 | Vertebra (0.01) | Klehkwahnnohm | Northern Georgia Strait | ~250BP | Hapd6 | Hapc4 |
| CP08 | Vertebra (0.02) | Klehkwahnnohm | Northern Georgia Strait | ~250BP | Hapd7 | Hapc5 |
| CP09 | Pro-otic (0.02) | Klehkwahnnohm | Northern Georgia Strait | ~250BP | Hapd8 | Hapc1 |
| CP10 | Vertebra(<0.01) | Klehkwahnnohm | Northern Georgia Strait | ~250BP | Hapd9 | Hapc6 |
| CP11 | Vertebra (0.01) | Klehkwahnnohm | Northern Georgia Strait | ~250BP | Hapd10 | Hapc1 |
| CP74 | Vertebra (0.009) | Kahkeeky | Northern Georgia Strait | 950-150BP | Hapd42 | Hapc1 |
| CP75 | Vertebra (0.008) | Kahkeeky | Northern Georgia Strait | 950-150BP | Hapd17 | Hapc10 |
| CP76 | Vertebra (0.011) | Kahkeeky | Northern Georgia Strait | 950-150BP | Failed | Failed |
| CP77 | Vertebra (0.013) | Kahkeeky | Northern Georgia Strait | 950-150BP | Hapd43 | Hapc15 |
| CP78 | Vertebra (0.007) | Kahkeeky | Northern Georgia Strait | 950-150BP | Failed | Failed |
| CP79 | Vertebra (0.004) | Kahkeeky | Northern Georgia Strait | 950-150BP | Failed | Failed |
| CP80 | Vertebra (0.006) | Kahkeeky | Northern Georgia Strait | 950-150BP | Failed | Failed |
| CP81 | Vertebra (0.003) | Kahkeeky | Northern Georgia Strait | 950-150BP | Hapd5 | Hapc1 |
| CP82 | Vertebra (0.003) | Kahkeeky | Northern Georgia Strait | 950-150BP | Failed | Failed |
| CP83 | Vertebra (0.003) | Kahkeeky | Northern Georgia Strait | 950-150BP | Failed | Failed |
| CP84 | Vertebra (0.003) | Kahkeeky | Northern Georgia Strait | 950-150BP | Hapd23 | Hapc1 |
| CP85 | Vertebra (0.014) | Kahkeeky | Northern Georgia Strait | 950-150BP | Hapd5 | Hapc1 |
| CP86 | Vertebra (0007) | Kahkeeky | Northern Georgia Strait | 950-150BP | Hapd3 | Hapc1 |
| CP52 | Vertebra (0.017) | 131T, Broken Group Islands Unit, PRNPR, Barkley Sound | West Coast of Vancouver Island | 1000-2000BP | Hapd12 | Hapc2 |
| CP53 | Vertebra (0.006) | 131T, Broken Group Islands Unit, PRNPR, Barkley Sound | West Coast of Vancouver Island | 1000-2000BP | Hapd30 | Hapc9 |
| CP54 | Vertebra (0.009) | 131T, Broken Group Islands Unit, PRNPR, Barkley Sound | West Coast of Vancouver Island | 1000-2000BP | Hapd31 | Hapc9 |
| CP55 | Vertebra (0.005) | 83T, Broken Group Islands Unit, PRNPR, Barkley Sound | West Coast of Vancouver Island | 500-800BP | Hapd32 | Hapc1 |
| CP56 | Vertebra (0.009) | 83T, Broken Group Islands Unit, PRNPR, Barkley Sound | West Coast of Vancouver Island | 500-800BP | Hapd33 | Hapc1 |
| CP57 | Vertebra (0.011) | 83T, Broken Group Islands Unit, PRNPR, Barkley Sound | West Coast of Vancouver Island | 500-800BP | Hapd12 | Hapc2 |
| CP58 | Vertebra (0.010) | 83T, Broken Group Islands Unit, PRNPR, Barkley Sound | West Coast of Vancouver Island | 500-800BP | Hapd34 | Hapc15 |
| CP59 | Vertebra (0.009) | 83T, Broken Group Islands Unit, PRNPR, Barkley Sound | West Coast of Vancouver Island | 500-800BP | Hapd35 | Hapc16 |
| CP60 | Vertebra (0.009) | 83T, Broken Group Islands Unit, PRNPR, Barkley Sound | West Coast of Vancouver Island | 500-800BP | Hapd36 | Hapc17 |
| CP61 | Vertebra (0.016) | 83T, Broken Group Islands Unit, PRNPR, Barkley Sound | West Coast of Vancouver Island | 500-250BP | Hapd37 | Hapc1 |
| CP62 | Vertebra (0.012) | 83T, Broken Group Islands Unit, PRNPR, Barkley Sound | West Coast of Vancouver Island | 500-250BP | Hapd29 | Hapc1 |
| CP63 | Vertebra (0.008) | 83T, Broken Group Islands Unit, PRNPR, Barkley Sound | West Coast of Vancouver Island | 500-250BP | Hapd24 | Hapc1 |
| CP64 | Vertebra (0.015) | 83T, Broken Group Islands Unit, PRNPR, Barkley Sound | West Coast of Vancouver Island | 500-250BP | Hapd6 | Hapc1 |
| CP65 | Vertebra (0.009) | 83T, Broken Group Islands Unit, PRNPR, Barkley Sound | West Coast of Vancouver Island | 500-250BP | Hapd38 | Hapc1 |
| CP66 | Vertebra (0.005) | 83T, Broken Group Islands Unit, PRNPR, Barkley Sound | West Coast of Vancouver Island | 500-250BP | Hapd39 | Hapc1 |
| CP67 | Vertebra (0.013) | 83T, Broken Group Islands Unit, PRNPR, Barkley Sound | West Coast of Vancouver Island | 250-100BP | Hapd35 | Hapc16 |
| CP68 | Vertebra (0.012) | 83T, Broken Group Islands Unit, PRNPR, Barkley Sound | West Coast of Vancouver Island | 250-100BP | Hapd40 | Hapc18 |
| CP69 | Vertebra (0.005) | 83T, Broken Group Islands Unit, PRNPR, Barkley Sound | West Coast of Vancouver Island | 250-100BP | Hapd24 | Hapc1 |
| CP70 | Vertebra (0.005) | 83T, Broken Group Islands Unit, PRNPR, Barkley Sound | West Coast of Vancouver Island | 250-100BP | Hapd7 | Hapc9 |
| CP71 | Vertebra (0.007) | 83T, Broken Group Islands Unit, PRNPR, Barkley Sound | West Coast of Vancouver Island | 250-100BP | Hapd17 | Hapc10 |
| CP72 | Vertebra (0.006) | 83T, Broken Group Islands Unit, PRNPR, Barkley Sound | West Coast of Vancouver Island | 250-100BP | Failed | Failed |
| CP73 | Vertebra (0.007) | 83T, Broken Group Islands Unit, PRNPR, Barkley Sound | West Coast of Vancouver Island | 250-100BP | Hapd41 | Hapc1 |
| CP12 | Vertebra (0.01) | Tum-tumay-whueton | Burrard Inlet | <3000BP | Failed | Failed |
| CP13 | Vertebra (<0.01) | Tum-tumay-whueton | Burrard Inlet | <3000BP | Hapd6 | Hapc1 |
| CP14 | Vertebra (<0.01) | Tum-tumay-whueton | Burrard Inlet | <3000BP | Hapd5 | Hapc1 |
| CP15 | Vertebra (<0.01) | Tum-tumay-whueton | Burrard Inlet | <3000BP | Failed | Hapc7 |
| CP16 | Vertebra (0.01) | Tum-tumay-whueton | Burrard Inlet | <3000BP | Hapd12 | Hapc2 |
| CP17 | Vertebra (<0.01) | Tum-tumay-whueton | Burrard Inlet | <3000BP | Hapd13 | Hapc1 |
| CP18 | Vertebra (<0.01) | Tum-tumay-whueton | Burrard Inlet | <3000BP | Failed | Hapc8 |
| CP19 | Vertebra (0.01) | Tum-tumay-whueton | Burrard Inlet | <3000BP | Hapd9 | Hapc6 |
| CP20 | Vertebra (<0.01) | Tum-tumay-whueton | Burrard Inlet | <3000BP | Hapd12 | Hapc2 |
| CP21 | Vertebra (<0.01) | Tum-tumay-whueton | Burrard Inlet | <3000BP | Hapd14 | Hapc1 |
| CP22 | Vertebra (<0.01) | Tum-tumay-whueton | Burrard Inlet | <3000BP | Hapd15 | Failed |
| CP23 | Vertebra (<0.01) | Tum-tumay-whueton | Burrard Inlet | <3000BP | Hapd7 | Hapc9 |
| CP24 | Vertebra (<0.01) | Tum-tumay-whueton | Burrard Inlet | <3000BP | Hapd15 | Hapc7 |
| CP25 | Vertebra (<0.01) | Tum-tumay-whueton | Burrard Inlet | <3000BP | Hapd7 | Hapc9 |
| CP26 | Vertebra (<0.01) | Tum-tumay-whueton | Burrard Inlet | <3000BP | Hapd16 | Hapc1 |
| CP27 | Vertebra (<0.01) | Tum-tumay-whueton | Burrard Inlet | <3000BP | Hapd17 | Hapc10 |
| CP28 | Vertebra (<0.01) | Tum-tumay-whueton | Burrard Inlet | <3000BP | Hapd18 | Hapc1 |
| CP29 | Vertebra (<0.01) | Tum-tumay-whueton | Burrard Inlet | <3000BP | Hapd12 | Hapc2 |
| CP30 | Vertebra (<0.01) | Tum-tumay-whueton | Burrard Inlet | <3000BP | Hapd7 | Hapc9 |
| CP31 | Vertebra (<0.01) | Tum-tumay-whueton | Burrard Inlet | <3000BP | Hapd19 | Hapc11 |
| CP32 | Vertebra (<0.01) | Tum-tumay-whueton | Burrard Inlet | <3000BP | Hapd7 | Hapc9 |
| CP33 | Vertebra (0.01) | Tum-tumay-whueton | Burrard Inlet | <3000BP | Hapd12 | Hapc2 |
| CP34 | Vertebra (<0.01) | Tum-tumay-whueton | Burrard Inlet | <3000BP | Hapd12 | Hapc2 |
| CP35 | Vertebra (<0.01) | Tum-tumay-whueton | Burrard Inlet | <3000BP | Hapd12 | Hapc2 |
| CP36 | Vertebra (<0.01) | Tum-tumay-whueton | Burrard Inlet | <3000BP | Hapd20 | Failed |
| CP37 | Vertebra (<0.01) | Tum-tumay-whueton | Burrard Inlet | <3000BP | Hapd21 | Hapc13 |
| CP38 | Vertebra (<0.01) | Tum-tumay-whueton | Burrard Inlet | <3000BP | Hapd15 | Hapc7 |
| CP39 | Vertebra (<0.01) | Tum-tumay-whueton | Burrard Inlet | <3000BP | Hapd22 | Hapc12 |
| CP40 | Vertebra (<0.01) | Tum-tumay-whueton | Burrard Inlet | <3000BP | Hapd23 | Hapc1 |
