# Supplementary material for: High Potential for Using DNA from Ancient Herring Bones to Inform Modern Fisheries Management and Conservation
Source: PLoS One. 2012 Nov 30;7(11):e51122. doi: 10.1371/journal.pone.0051122 (PMC3511397; doi:10.1371/journal.pone.0051122)
Supplement: Table S2 — Modern and ancient herring haplotype ( h ) and nucleotide (π) diversities [92] based on 350 bp mtDNA D-loop fragment. (DOCX) [file pone.0051122.s005.docx]

**Table S2. Modern and ancient herring haplotype (*h*) and nucleotide (π) diversities [92] based on 350bp mtDNA D-loop fragment.**

| **Sampling Sites** | **Sample codes** | **n** | **Haplotypes** | ***h*** | **(SD)** | **π** | **(SD)** |
| --- | --- | --- | --- | --- | --- | --- | --- |
| Peter the Great Bay | Pv | 68 | 35 | 0.946 | (0.015) | 0.01134 | (0.00076) |
| Vzmorye | Vz | 49 | 28 | 0.948 | (0.018) | 0.01321 | (0.00082) |
| Krasnogorsk | Oz | 48 | 19 | 0.929 | (0.017) | 0.01340 | (0.00094) |
| Aleksandrovsk* | AL | 50 | 27 | 0.953 | (0.016) | 0.01330 | (0.00104) |
| Taui Bay | AR | 52 | 27 | 0.929 | (0.021) | 0.01424 | (0.00122) |
| Gizhigin Bay | GI | 52 | 27 | 0.925 | (0.024) | 0.01493 | (0.00093) |
| Ilpir | Kk | 16 | 14 | 0.983 | (0.028) | 0.01422 | (0.00211) |
| Bering Sea | BS | 45 | 17 | 0.879 | (0.030) | 0.01000 | (0.00087) |
| Port Moller | PM | 47 | 22 | 0.921 | (0.025) | 0.01102 | (0.00079) |
| Simpson Sound | SP | 48 | 34 | 0.977 | (0.011) | 0.02680 | (0.00128) |
| Yakutat Bay* | YB | 48 | 37 | 0.987 | (0.008) | 0.02711 | (0.00128) |
| North Sitka | NS | 42 | 29 | 0.980 | (0.010) | 0.02459 | (0.00150) |
| South Sitka | SS | 47 | 38 | 0.989 | (0.007) | 0.02748 | (0.00110) |
| Selwyn Inlet | SI | 43 | 33 | 0.983 | (0.010) | 0.02555 | (0.00144) |
| Knight Inlet* | KI | 46 | 33 | 0.984 | (0.008) | 0.02728 | (0.00108) |
| Nootka Sound* | NO | 46 | 31 | 0.964 | (0.016) | 0.02616 | (0.00126) |
| Portage Inlet* | PI | 44 | 19 | 0.810 | (0.060) | 0.02067 | (0.00222) |
| Columbia River | CR | 53 | 41 | 0.991 | (0.005) | 0.02767 | (0.00126) |
| Tomales Bay | TB | 47 | 34 | 0.978 | (0.012) | 0.02525 | (0.00125) |
| San Francisco Bay | SF | 44 | 33 | 0.973 | (0.016) | 0.02401 | (0.00143) |
| **Southeast Alaska** | **CP42-51** | **9** | **9** | **1.000** | **(0.052)** | **0.02460** | **(0.00239)** |
| **Northern Georgia Strait** | **CP1-11, CP74-83** | **18** | **14** | **0.961** | **(0.034)** | **0.02211** | **(0.00326)** |
| **Burrard Inlet** | **CP13-40** | **26** | **16** | **0.926** | **(0.035)** | **0.02451** | **(0.00162)** |
| **West Coast of Vancouver Island** | **CP52-73** | **21** | **18** | **0.986** | **(0.019)** | **0.02517** | **(0.00193)** |
| **Total** |  | **1009** | **335** | **0.981** | **(0.001)** | **0.02716** | **(0.00029)** |

Note: Ancient samples in bold font; modern sites in regular font (from Liu et al.[19] main text)
* Populations included in comparisons of modern and ancient D-loop haplotypes in Fig 2 (main text)
