# Supplementary material for: High Potential for Using DNA from Ancient Herring Bones to Inform Modern Fisheries Management and Conservation
Source: PLoS One. 2012 Nov 30;7(11):e51122. doi: 10.1371/journal.pone.0051122 (PMC3511397; doi:10.1371/journal.pone.0051122)
Supplement: Table S3 — Modern and ancient herring haplotype ( h ) and nucleotide (π) diversities [92] based on 235 bp mtDNA cytb fragment. (DOCX) [file pone.0051122.s006.docx]

**Table S3. Modern and ancient herring haplotype (*h*) and nucleotide (π) diversities [92] based on 235bp mtDNA cytb fragment.**

| **Sampling Sites** | **Sample codes** | **n** | **Haplotypes** | ***h*** | **(SD)** | **π** | **(SD)** |
| --- | --- | --- | --- | --- | --- | --- | --- |
| Bering Sea | BS | 22 | 3 | 0.177 | (0.106) | 0.00114 | (0.00073) |
| Port Moller | PM | 24 | 3 | 0.239 | (0.113) | 0.00139 | (0.00072) |
| Simpson Sound | SP | 24 | 11 | 0.819 | (0.073) | 0.00665 | (0.00143) |
| Yakutat Bay | YB | 24 | 7 | 0.685 | (0.092) | 0.0492 | (0.00104) |
| Knight Inlet | KI | 14 | 7 | 0.813 | (0.094) | 0.00636 | (0.00137) |
| Portage Inlet | PI | 8 | 4 | 0.786 | (0.113) | 0.00578 | (0.00104) |
| Columbia River | CR | 23 | 11 | 0.735 | 0.100 | 0.00464 | 0.00097 |
| **Southeast Alaska** | **CP42-51** | **8** | **6** | **0.929** | **(0.084)** | **0.00699** | **(0.00146)** |
| **Northern Georgia Strait** | **CP1-11, CP74-83** | **18** | **8** | **0.641** | **(0.130)** | **0.00464** | **(0.00143)** |
| **Burrard Inlet** | **CP13-40** | **26** | **10** | **0.862** | **(0.040)** | **0.00649** | **(0.00117)** |
| **West Coast of Vancouver Island** | **CP52-73** | **21** | **8** | **0.762** | **(0.088)** | **0.00596** | **(0.00131)** |
| **Total** |  | **119** | **22** | **0.783** | **(0.035)** | **0.00586** | **(0.00054)** |

Note: Ancient samples in bold font; modern sites in regular font (from Liu et al.[19] main text)
