# Supplementary material for: High Potential for Using DNA from Ancient Herring Bones to Inform Modern Fisheries Management and Conservation
Source: PLoS One. 2012 Nov 30;7(11):e51122. doi: 10.1371/journal.pone.0051122 (PMC3511397; doi:10.1371/journal.pone.0051122)
Supplement: Table S4 — Microsatellite analyses. (DOCX) [file pone.0051122.s007.docx]

**Table S4. Microsatellite analyses.**

| **Sample Name** | **Cpa103c (179-247bp)** | | CPA 4b (97-108bp) | | CHA 113a (100-150bp) | | CHA 113a Repeat | |
| --- | --- | --- | --- | --- | --- | --- | --- | --- |
| CP19a | 198 | 226 | - | - | 116 | 140 | 118 | 140 |
| CP22a | - | - | - | - | - | - | - | - |
| CP23a | 218 | 218 | 120 | 120 | 110 | 110 | 110 | 110 |
| CP24a | - | - | - | - | 134 | 136 | 132 | 134 |
| CP25a | 218 | 218 | 120 | 128 | 110 | 138 | 110 | 110 |
| CP26a | 186 | 186 | 124 | 144 | 110 | 126 | 110 | 124 |
| CP31a | 210 | 246 | 128 | 180 | 108 | 134 | 108 | 134 |
| CP32a | 206 | 218 | 144 | 148 | 110 | 110 | 110 | 156 |
| CP33a | 206 | 206 | 144 | 144 | 110 | 110 | - | - |
| CP34a | 206 | 206 | 144 | 144 | 110 | 110 | 110 | 146 |
| CP52a | 210 | 242 | 136 | 160 | 110 | 130 | 110 | 130 |
| CP53a | - | - | 120 | 132 | 106 | 106 | 106 | 106 |
| CP54a | 210 | 218 | 136 | 136 | 140 | 142 | 140 | 144 |
| CP55a | 198 | 222 | 128 | 140 | 108 | 130 | 108 | 130 |
| CP56a | 218 | 218 | 144 | 148 | 108 | 128 | 108 | 126 |
| CP57a | 218 | 218 | 136 | 136 | 116 | 118 | 116 | 120 |
| CP61a | 218 | 218 | - | - | 112 | 132 | 112 | 132 |
| CP62a | - | - | 136 | 160 | 108 | 130 | 108 | 128 |
| CP63a | 206 | 206 | 128 | 160 | 126 | 128 | 126 | 126 |
| CP67a | - | - | - | - | 118 | 118 | 102 | 102 |
| CP68a | 210 | 210 | 144 | 160 | 130 | 140 | 130 | 140 |
| CP69a | - | - | 152 | 172 | 138 | 140 | 140 | 140 |
