# Supplementary material for: High Potential for Using DNA from Ancient Herring Bones to Inform Modern Fisheries Management and Conservation
Source: PLoS One. 2012 Nov 30;7(11):e51122. doi: 10.1371/journal.pone.0051122 (PMC3511397; doi:10.1371/journal.pone.0051122)
Supplement: Table S5 — Modern and ancient herring SNP genotypes. (DOCX) [file pone.0051122.s008.docx]

**Table S5. Modern and ancient herring SNP genotypes.**

| **Sample Name** | **Repeat 1** | **Repeat 2** | Repeat 3 | **Consensus SNP Call** | **Average Ct value** |
| --- | --- | --- | --- | --- | --- |
| CM1 | GG | GG | n/a | **GG** | 24.8 |
| CM2 | GT | GT | n/a | **GT** | 24.7 |
| CM5 | GT | GT | n/a | **GT** | 25.1 |
| CM6 | GT | GT | n/a | **GT** | 24.8 |
| CM7 | GT | GT | n/a | **GT** | 25.8 |
| CM12 | GG | GG | n/a | **GG** | 25.5 |
| CM13 | GG | GG | n/a | **GG** | 25.7 |
| CM15 | GG | GG | n/a | **GG** | 25.1 |
| CM22 | GG | GG | n/a | **GG** | 25.2 |
| CM23 | TT | TT | n/a | **TT** | 25.4 |
| CM24 | TT | TT | n/a | **TT** | 24.2 |
| CM38 | GG | GG | n/a | **GG** | 24.8 |
| CM39 | GT | GT | n/a | **GT** | 25.7 |
| CM40 | GT | GT | n/a | **GT** | 25.4 |
| CM43 | TT | TT | n/a | **TT** | 24.4 |
| CM44 | GT | GT | n/a | **GT** | 25.6 |
| CM45 | GT | GT | n/a | **GT** | 25.9 |
| CM49 | UND | UND | n/a | **-** | 35.3 |
| CM50 | TT | TT | n/a | **TT** | 23.6 |
| CM51 | GG | GG | n/a | **GG** | 24.5 |
| CM55 | GG | GG | n/a | **GG** | 24.9 |
| CM56 | GG | GG | n/a | **GG** | 24.7 |
| CM57 | GT | GT | n/a | **GT** | 25.7 |
| CM61 | NOAMP | NOAMP | n/a | **-** | - |
| CM62 | GT | GT | n/a | **GT** | 24.2 |
| CM63 | GT | GT | n/a | **GT** | 32.1 |
| CM66 | GT | GT | n/a | **GT** | 26.0 |
| CM67 | GT | GT | n/a | **GT** | 26.7 |
| CM71 | TT | TT | n/a | **TT** | 25.3 |
| CM72 | TT | TT | n/a | **TT** | 24.9 |
| CP2 | GT | TT | GT | **GT** | 38.9 |
| CP9 | TT | TT | TT | **TT** | 33.0 |
| CP14 | GT | GT | GT | **GT** | 36.8 |
| CP19 | GG | GG | GG | **GG** | 35.1 |
| CP22 | NOAMP | NOAMP | NOAMP | **-** | - |
| CP23 | GG | GG | GG | **GG** | 37.8 |
| CP24 | NOAMP | NOAMP | GG | **-** | 38.6 |
| CP25 | GG | GG | GG | **GG** | 37.6 |
| CP26 | TT | TT | TT | **TT** | 33.7 |
| CP31 | GG | GG | GG | **GG** | 34.5 |
| CP32 | GG | GT | GT | **GT** | 37.0 |
| CP33 | TT | GT | UND | **-** | 38.2 |
| CP34 | GT | TT | GT | **GT** | 38.1 |
| CP52 | UND | UND | UND | **-** | 35.3 |
| CP53 | UND | GG | GG | **GG** | 39.3 |
| CP54 | GT | UND | UND | **-** | 36.2 |
| CP55 | GT | UND | UND | **-** | 36.5 |
| CP56 | UND | GT | GT | **GT** | 36.5 |
| CP57 | GG | GG | GG | **GG** | 33.3 |
| CP59 | GG | GG | GG | **GG** | 32.7 |
| CP61 | GT | GT | GT | **GT** | 35.2 |
| CP62 | GG | GG | GG | **GG** | 37.1 |
| CP63 | GG | GG | GG | **GG** | 34.9 |
| CP67 | NOAMP | NOAMP | TT | **-** | 39.2 |
| CP68 | GG | GG | GG | **GG** | 36.0 |
| CP69 | GT | GT | GT | **GT** | 36.2 |
| CP73 | GT | GT | GT | **GT** | 36.8 |
| CP75 | TT | TT | n/a | **TT** | 35.4 |
| CP79 | UND | NOAMP | UND | **-** | 47.7 |
| CP84 | GG | GG | GG | **GG** | 38.4 |

Note: NOAMP indicates that no amplification was detected, UND indicates that the amplification occurred but the genotype was undetermined and n/a indicates that the sample was not repeated a third time.
