# Supplementary material for: High Potential for Using DNA from Ancient Herring Bones to Inform Modern Fisheries Management and Conservation
Source: PLoS One. 2012 Nov 30;7(11):e51122. doi: 10.1371/journal.pone.0051122 (PMC3511397; doi:10.1371/journal.pone.0051122)
Supplement: Table S6 — Cytb and D-loop Clupea mtDNA PCR amplification primers. (DOCX) [file pone.0051122.s009.docx]

**Table S6. Cytb and D-loop *Clupea* mtDNA PCR amplification primers.**

| **Primer Name^a^** | **Sequence (5' to 3')** | **Coordinates^b^** | **Bases** | **Tm (°C)^c^** |
| --- | --- | --- | --- | --- |
| CP-F6-DL | CCACCCCTAACTCCCAAAGC | 15607-15626 | 20 | 58.1 |
| CP-F130-DL | GTCTTTCCATAACTGCAATATCAC | 15730-15753 | 24 | 52.0 |
| CP-R425-DL | GTGATTTTAATGATRAGCTCG | 16004-16024 | 21 | 48.2 |
| CP-R289-DL | CATSCATGTATCAATACATAGGTAC | 15865-15889 | 25 | 51.2 |
| CP-F30-CB | GCC TAC GAA AAA CCC ACC C | 14380-14398 | 19 | 56.4 |
| CP-R328-CB | TAG TCC TCG GGC GAT ATG TG | 14659-14678 | 20 | 56.1 |

^a^ F and R in the primer name denote forward and reverse primers, respectively; DL indicates d-loop primers, while CB indicates cytb primers.

^b^ Coordinates, numbered according to the GenBank accession NC009578.

^c^ Tm calculated based on 50mM NaCl
