# Supplementary material for: High Potential for Using DNA from Ancient Herring Bones to Inform Modern Fisheries Management and Conservation
Source: PLoS One. 2012 Nov 30;7(11):e51122. doi: 10.1371/journal.pone.0051122 (PMC3511397; doi:10.1371/journal.pone.0051122)
Supplement: Table S7 — Reporter sequence and primers for SNP assay. (DOCX) [file pone.0051122.s010.docx]

**Table S7. Reporter sequence and primers for SNP assay.**

| Assay Name | Sequence |
| --- | --- |
| Cpa_11961_c04 | TGAGGAGACCCAGTGAGGTCTGCTGGACCACACCCCCTCTGTGTTATGGCACAGGGAGTGGATCTATG[G/T]ATGATGGATGGGAAGGCATTTGTCTATCTTTGTCAACGCCTGATG |
| Forward primer | GGACCACACCCCCTCTGT |
| Reverse primer | TCAGGCGTTGACAAAGATAGACAAA |

Note: Highlighted area denotes reporter sequence (VIC dye binds the G allele, FAM dye binds the T allele); primer sequences listed 5' to 3';
